# Existing evidence of conceptual differences in research on climate change perceptions among smallholders? A systematic map

**DOI:** 10.1186/s13750-023-00321-2

**Published:** 2023-12-07

**Authors:** Lia Taruiap Troncarelli, Maíra Teixeira de Ataide, Carla Morsello

**Affiliations:** 1 Institute of Energy and the Environment, University of São Paulo, Av. Prof. Luciano Gualberto, 1289 - Vila Universitária, São Paulo, 05508-900 Brazil; 2 School of Arts, Sciences and Humanities, University of São Paulo, Rua Arlindo Béttio, 1000 - Ermelino Matarazzo, São Paulo, 03828-000 Brazil

**Keywords:** Climate change awareness, Construct, Experience, Global warming, Indigenous people, Public perception, Smallholders, Small-scale societies, Risk perception, Traditional knowledge

## Abstract

**Background:**

Climate change is having adverse effects on the livelihoods of small-scale populations, particularly in relation to their subsistence practices. Scientific literature widely acknowledges that smallholders must first perceive climate changes to take necessary precautions and adapt to the new conditions. However, variations exist in the terminology used across the literature, and in how it conceptualizes these perceptions. This variation complicates understanding of the literature and hinders empirical evidence comparisons. Therefore, in this review, we systematically mapped the literature considering variations in the concept's usage across different thematic areas. Our goal was to provide a comprehensive overview of the current state of the literature on smallholder climate change perceptions.

**Methods:**

In our systematic map, we adhered to the Collaboration for Environmental Evidence guidelines. We searched the literature adopting English terms and using five electronic databases of scientific publications (Web of Science Core Collection, Scopus, BASE–Bielefeld Academic Search Engine, PubMed, and Science Direct Elsevier). We then screened the retrieved articles' titles, abstracts, and full texts according to predefined eligibility criteria. Articles meeting the eligibility criteria were chosen for full reading, data extraction, and coding, utilizing a prepared codebook. No validity appraisal occurred in this selection. A database containing coded metadata for all studies is accessible for reference.

**Review findings:**

After screening 5358 articles (titles and abstracts), we identified and thoroughly reviewed 361 eligible articles at full text to map the usage of the climate change perception concept. Among these, 73 articles provided explicit definitions of perception, falling into seven categories: risk perception, perception based on psychological constructs and sensory stimuli, awareness, prior experience, observation of climate variables, beliefs, and uncertainties or threats. Implicit definitions of perception with various constructs were found, including those rooted in Cognitive Psychology, awareness, risk perception, traditional knowledge, beliefs, concerns about climate change, experiences of exposure to its effects, attitudes, worldviews, and scientific knowledge. Articles usually address multiple topics. Notably, 88% of the articles did not present any theory throughout their content. Geographically, Africa and Asia were the most frequently studied continents, with more focus on non-indigenous small-scale populations than indigenous ones.

**Conclusions:**

In conclusion, the perception concept exhibits an interdisciplinary nature. Therefore, fostering continuous dialogue among diverse disciplines is imperative to establishing an interdisciplinary definition of the term. An in-depth understanding of the perception concept is essential, as its absence can result in erroneous conclusions, limited adaptation strategies, and a lack of awareness among small-scale populations regarding climate change impacts. Misconceptions about this concept can lead to ineffective policies, further endangering vulnerable populations. Defining the concept and its constructs facilitates article comparisons. Without this definition, meaningful comparisons become unfeasible. Moreover, the absence of proper perception definitions poses challenges for small-scale populations, researchers, and stakeholders in developing effective, efficient, and flexible adaptations over time. Perception is the first step in incorporating adaptation strategies and must be translated into policies to address climate change impacts efficiently.

**Supplementary Information:**

The online version contains supplementary material available at 10.1186/s13750-023-00321-2.

## Background

Human-induced climate change has impacted every part of the globe, with human activities leading to warming the Earth's soils, oceans, and atmosphere over the past two millennia. In addition to rising temperatures, since the 1950s, the likelihood of extreme events like heatwaves, severe droughts, heavy rainfall, flooding, and tropical cyclones has increased [1]. Looking ahead, the Intergovernmental Panel on Climate Change (IPCC) anticipates that global warming will unavoidably surpass the 1.5 to 2 °C threshold in the twenty-first century unless there are substantial reductions in carbon dioxide and other greenhouse gas emissions in the coming decades [1, 2]. These changes in climate pose risks to various aspects of human life, including food security, health, access to water resources, and overall economic activities. As a result, human livelihoods, especially those of vulnerable populations in developing nations, are under threat [1]. Projections suggest that by 2030, climate change could push approximately 132 million people into poverty [3].

The livelihoods of small-scale rural communities, including indigenous groups, are already experiencing detrimental effects due to global warming [4]. This is primarily attributed to their heavy dependence on natural resources as a means of sustaining their way of life [5]. The impacts of climate change profoundly affect the lives and culture of small-scale populations. Notably, the increased frequency of extreme weather events, such as prolonged floods and droughts, has adversely impacted the agriculture of diverse small-scale populations worldwide [6–9], resulting in food insecurity [10, 11] and heightened vulnerability to diseases [12]. This, in turn, adversely affects both the physical and mental health of these populations [13, 14]. Furthermore, shifts in water temperature, water levels, water color, or turbidity have impacted fishing, compromising the livelihoods of populations dependent on this means of subsistence [15, 16]. In terms of hunting, rising temperatures, changes in rainfall patterns, and shifts in ecosystems have resulted in the scarcity of animals in specific regions, negatively impacting hunting-dependent communities [16, 17]. In addition to immediate impacts, climate change has prompted population migration due to the potential deterioration of living conditions for these communities [18, 19].

On the other side, small-scale populations have a long-term connection with their natural environments, which may allow them to detect, understand, and act to mitigate the adverse effects of environmental changes [20]. Detailed traditional knowledge, which developed from smallholders’ close interaction with the environment, is particularly important in this endeavor [21].

Nonetheless, to achieve these goals, small-scale communities must initially become aware of shifts in the climate. The ability to perceive these changes is crucial because small-scale populations are more inclined to take preventive measures, respond to ongoing negative effects, and capitalize on potential advantages when aware of these alterations [22, 23]. This process, often referred to as adaptation strategies [24], only commences once a population acknowledges the changes and possesses information regarding their probable consequences, whether accurate or not [25].

Apart from the significance of the population's direct awareness of climate change and its consequences, it is imperative to comprehend the mechanisms underlying this process for two principal reasons. Firstly, pertinent and useful traditional knowledge in this regard may currently be overlooked in the scientific literature. Secondly, insights derived from local knowledge can provide valuable information about climate conditions in regions where meteorological data and monitoring stations are insufficient [26]. In essence, it is paramount to investigate how individuals within small communities perceive climate change and the timing and methods of their perception. Scientists have acknowledged this significance, leading to numerous studies on this subject [27, 28].

Nevertheless, many studies on climate change fail to incorporate a specific or at least a transparent definition of perception (e.g., [29–35]). While the term may vary in its interpretations, even within the same research domain, a shared aspect in most definitions of perception is its engagement with the examination of and individual’s sensory information. Therefore, when cognitive psychologists discuss perceptions, they typically address the fundamental cognitive procedures associated with interpreting sensory information received from individuals' senses [36]. Nevertheless, within the literature on climate change that defines “perceptions” (e.g., [37–41]), the conceptualizations employed are varied. They encompass not just sensory perceptions (e.g., [38, 42–44]), but also individuals’ subjective interpretations (e.g., [45]), awareness or the understanding of environmental experiences (e.g., [26, 34]), beliefs regarding ongoing changes (e.g., [39, 41]), and past experiences linked to climate fluctuations (e.g., [41]).

The diverse range of definitions for climate change perception adopted presents three key challenges that hinder the comparison and synthesis of the scientific literature. Firstly, numerous thematic areas encompass incorporate the concept of climate change perception. However, the precise meaning of the term’s usage remains unclear. This lack of standardization within and across areas, but also within a single study, makes it difficult to synthesize and compare results, given the presentation of different interpretations. For example, the concept is integrated into studies concerning adaptation strategies [23], traditional or local knowledge [32, 46] and its comparison with actual meteorological data [45, 47], cognitive biases [30], and the identification of factors that explain perceptions [48]. Despite that, the concept varied across and within these areas.

Secondly, although cognitive perceptions pertain to individuals, studies diverge in their choice of sample units for investigating the phenomenon, which may refer to individuals (e.g., [49–52]), households (e.g., [53–56]), individual and household (e.g., [33, 39, 57–59]) or even communities and large-scale populations (e.g., [60]). This factor adds another layer of confusion in interpreting and comparing results.

Thirdly, studies employ diverse investigation methods [61], including qualitative (e.g., [62–65]), quantitative (e.g., [66–69]), or mixed approaches (e.g., [70–73]). Again, these methodological differences have implications for how results are interpreted and for the definition of the perception concept itself.

Thus, this conceptual map summarizes information on various interpretations of climate change perception, enhancing our understanding of its variability across different domains and fostering the standardization of terminology in this field. Consequently, this article aims to streamline and enhance the reliability of information synthesis and comparisons within the existing empirical literature. These evaluations hold significance in formulating and implementing adaptation policies, especially within small-scale societies. In this regard, this article also strives to pinpoint areas where further assessments and syntheses on climate change perception are necessary, thereby assisting in identifying knowledge gaps for future research.

### Theoretical framework on perception

The study of human perception is primarily conducted within the realm of Cognitive Psychology [74, 75], a scientific discipline that began to take shape in the 1950s but was formally recognized as a distinct field with its own methods in the 1970s [75]. Beyond the study of human perceptions, this field explores various other mental processes, including memory, language, attention, problem-solving, consciousness, emotion, reasoning [76], learning, and motivation [75].

In the realm of Cognitive Psychology, perception is defined as a series of intricate steps whereby an individual processes information derived from environmental stimuli and assigns meaning to them [75]. Perception encompasses sensory analysis [77], because it is from the sensations detected through sensory receptors (such as ears, nose, tongue, skin, and eyes) that individuals recognize, interpret, and organize information [78].

Within the domain of Cognitive Psychology, the theories explaining how human perception functions can be categorized into two groups: top–down and bottom–up theories [75].

Bottom-up theories, also known as direct perception theories, posit that stimuli and sensory information are the initial components of perception [77]. In this view, a person observes something, and the information gathered by sensory receptors is conveyed to the brain [75]. Consequently, individuals would rely on their senses (sight, hearing, smell, touch) when encountering extreme weather events and the consequences of climate change.

Top-down theories, also called constructive perception theories, propose that humans initially employ pre-existing knowledge stored in memory, already-existing expectations about what they are perceiving, cognitive processes, and, at times, prior experiences to shape their processing of sensory information [75]. Under this framework, what an individual perceives depends on his/her existing knowledge [76]. Individuals would first draw upon their existing knowledge to form an idea of climate change and its impacts, and only after observing it would they comprehend its true nature. This prior knowledge may originate from information acquired through (i) indirect exposure (such as reports from other people or access to the media), (ii) direct exposure (first-hand contact with the impacts of changes), (iii) traditional knowledge transmitted through generations, and (iv) scientific knowledge.

While these alternative theories are often presented as opposing viewpoints in the literature, they can actually complement each other because individuals are likely to employ both processes in perception [75] as they address different aspects of perception [76]. Both theories are relevant to the understanding of climate change perception, as the direct perception of sensory stimuli, combined with knowledge and prior experiences, contributes to an individual's understanding of climate change.

For perception to take place, two naturally occurring stimuli are necessary: distal and proximal. In the distal stimulus, a physical object or event is situated at some physical distance from the individual [75, 78]. The image of this object or event reaches the sensory receptors, known as the proximal stimulus. The proximal stimulus can be influenced by various factors, such as lighting conditions, color, viewing angle, alterations in shape [78], tactile information, sound waves, olfactory or gustatory stimulation, or chemical molecules, with variations depending on each receptor [75]. As a result, perception emerges when an individual elaborates on what is being sensory perceived, effectively reflecting the external world [75].

In the context of climate change (based on bottom-up theory), perception can result from the combination of various sensations and stimuli, including what an individual can visually observe from a certain physical distance. This occurs, for instance, when one observes extreme weather events such as floods, heavy rainfalls, severe droughts, wildfires, and numerous others. In addition to sight, tactile sensations produced by the event may also play a role. For example, during extreme heatwaves, the sensation of heat is intensified, while abrupt temperature drops tend to make cold sensations more pronounced. During floods, individuals can feel the water on their skin. The sound emitted by the event is another critical stimulus, including sounds like rain, noise from rising river levels, wind knocking down trees, or the crackling of a forest fire. The sense of smell also contributes to perception; for instance, individuals may perceive the smell of burning trees or emitted polluting gases. Furthermore, based on top-down theory, individuals would also use experiences with adverse climate impacts, such as encountering heatwaves, extreme droughts, or floods, to perceive climate change. In addition to this perception, pre-existing memory and knowledge are incorporated, acquired directly through experiences with changes or indirectly through various sources of information (media, individual reports, and school).

While perception enables individuals to become aware of the impacts of climate change, adapt to them, and interact with the world, it can be constrained by at least four factors.

Firstly, many climate changes unfold slowly, at least when viewed from a non-geological perspective (e.g., the slowdown of ocean circulation, the disintegration of ice sheets, permafrost thaw) [79]. Consequently, humans may not readily discern what is occurring and, as a result, underestimate these changes because they cannot visually perceive climate change as an ongoing process [80]. Contributing to this limitation is the fact that humans only perceive a limited range of the electromagnetic spectrum (visible light), can only hear frequencies between 20 and 20,000 Hz, and are unable to perceive events that occur too rapidly or too slowly [78].

A second factor to consider is that perception can be prone to inaccuracies, as physical phenomena can create optical illusions [78]. In other words, what we perceive through our sensory organs may not always align with reality [75].

Thirdly, human perception is selective because attention tends to be directed toward specific aspects while disregarding the surroundings. Consequently, our attention can be diverted when a more attention-grabbing event occurs [78]. Therefore, what is perceived through the senses is contingent on what captures our attention, is selected, and forms within the mind [75].

Lastly, various factors, such as the context [78, 81], individual and societal beliefs, cultural practices, and individual’s prior experiences, can influence one's perception of climate change [80].

## The objective of the review

The objective of this systematic mapping was to identify, categorize, and provide an overview of the existing evidence found in scientific literature concerning the diverse terms and ideas used to describe how people perceive climate change.

This review has focused on the literature of the last five years (2018 to 2022) regarding small-scale rural populations, including indigenous societies. We selected small-scale rural communities because these societies directly rely on natural resources, putting them at a higher risk of experiencing the negative impacts of climate change [5]. In addition, the perception of climate change can assist them in adapting their subsistence practices [82], suffering fewer potential adverse effects.

Our primary research question was, therefore:

*“What evidence exists on the alternative definitions of climate change perceptions adopted in the literature about small-scale populations of the last five years (2018 to 2022)?”*

The secondary research questions were:

*“How do the definitions of climate change perception vary and are interpreted across articles, according to their thematic areas, populations of interest, and geographical origin?*

*What constructs differentiate one definition from another? Are there similarities?”*

Constructs are theoretical concepts that help test hypotheses based on observable phenomena, defined as “a complex idea or concept formed from a synthesis of simpler ideas” [83].

The components of the primary question were as follows:

Population (P): Small communities inhabiting remote rural regions, composed of individuals who sustain their livelihoods through family labor and have limited to no capacity to generate excess production for commercial purposes [84].

Exposure (E): Climate change, including extreme events such as floods, heatwaves, intense droughts, heavy rainfalls, tropical cyclones, among others.

Outcome (O): Implicit of explicit definitions of climate change perceptions.

## Methods

This map was conducted according to the published protocol [85]. The map follows the Collaboration for Environmental Evidence (CEE) Guidelines and Standards for Evidence Synthesis [86], and conforms to the Reporting Standards for Systematic Evidence (ROSES) [87] (see Additional file 1).

### Deviations from the protocol

In this article, we deviated from our published protocol [85] as regards the time frame due to limited resources. Although the original protocol did not specify the review period, we confined it to a five-year timeframe (2018 to 2022). The review team (hereafter referred as team) acknowledges that this deviation may have introduced limitations to the results, as discussed toward the end of this article.

### Search for articles

Searches were conducted in five electronic scientific databases of publications: “Web of Science Core Collection” (WoS)”, “Scopus”, “BASE–Bielefeld Academic Search Engine”, “PubMed” and “Science Direct Elsevier”, between March 2022 and February 2023, seeking articles between 2018 and 2022. We selected these databases because they are extensive, cover a wide range of disciplines, and include peer-reviewed publications. We assumed that they would include the majority of publications related to climate change perceptions within the Environmental Sciences field. Moreover, all five chosen databases have established procedures to ensure the quality of published research.

#### Search terms and language

The search query for this review, which involved the use of Boolean operators (AND, OR) to combine key terms, consisted of English terms that fell into three distinct conceptual groups: (i) perception or awareness; (ii) climate change or global warming; and (iii) smallholders, including indigenous population. We employed an asterisk as a special character to account for variations in word endings and plurals, except when using BASE, Science Direct, and PubMed, as these platforms do not support this procedure. Only documents in English were reviewed because of (i) familiarity with this idiom and (ii) English is the universal scientific language (for more information, see protocol [83].

The team searched five databases by looking for the chosen terms within the article's title, abstract, or keywords. The final search strings are described below.

- *Scopus:* (TITLE-ABS-KEY ((("perception*" OR "local perspective*" OR "awareness") AND ("climat* chang*" OR "global warming" OR "chang* climat*" OR "climat* variabilit*" OR "climat* event*") AND ("indigenous*" OR "smallholder*" OR "small scale*" OR "livelihood*" OR "fisher*" OR "peasant*" OR "hunter*" OR "agricultur*" OR "forager*" OR "agropastoralist*" OR "horticultur*" OR "pastoralist*" OR “herder*” OR “small-island*”))))
- *WoS:* ALL FIELDS (("perception*" OR "local perspective*" OR "awareness") AND ("climat* chang*" OR "global warming" OR "chang* climat*" OR "climat* variabilit*" OR "climat* event*") AND ("indigenous*" OR "smallholder*" OR "small scale*" OR "livelihood*" OR "fisher*" OR "peasant*" OR "hunter*" OR "agricultur*" OR "forager*" OR "agropastoralist*" OR "horticultur*" OR "pastoralist*" OR “herder*” OR “small-island*”))
- *BASE:* Entire document: ("perception" OR "awareness") AND ("climate change" OR "global warming") AND ("indigenous" OR "smallholder" OR “small-island”)
- *Science Direct:* Title, abstract, keywords: (("perception" OR "awareness") AND ("climate change" OR "global warming") AND ("indigenous" OR "smallholder" OR “small-island”))
- *PubMed:* (("perception" OR "awareness") AND ("climate change" OR "global warming") AND ("indigenous" OR "smallholder" OR “small-island”))

#### Search sources and results

The team accessed the five databases using the University of São Paulo's institutional subscription via a Virtual Private Network (VPN) in Brazil. In Scopus and WoS, we applied two filters to the search fields: (i) we looked specifically for articles in the document type, and (ii) we narrowed it down to articles written in English. In BASE, we conducted a basic search, filtered the results by articles written in English, and categorized them as article contributions in the document type. We opted for an advanced search for Science Direct, filtering by title, abstract, or author-specified keywords, and entered the search string. In PubMed, we did not apply any filters. Our selection criteria were limited to articles that presented primary data. Books and book chapters were also omitted from the study because we could not ensure access to them. In summary, the following types of articles were also excluded: (i) documents in languages other than English and (ii) review articles, books, book chapters, conference papers, proceedings papers, conference reviews, editorials, letters, and data papers.

Prior to the screening process, we identified and removed duplicate articles retrieved from all five databases using an Excel*®* spreadsheet. Team members did not participate in decisions regarding inclusion and evaluation when an article was authored by any of them. Only one article met this criterion, corresponding to the published protocol, which was excluded from the database [85]. Thus, no team member was excluded from participating in any coding decisions.

The review team did not pursue further endeavors to acquire literature (i) in other languages and (ii) from non-academic (grey) literature, and (iii) consultations with experts or other stakeholders. Although this is a limitation of our map that may be advanced in further studies, our time and resources were restricted.

#### Estimating the comprehensiveness of the search

As described in our protocol [85], the comprehensiveness of the search was assessed using a list of 95 benchmark articles compiled by the team members. The final search string applied to the three databases yielded 94 articles (representing 99% of the pre-defined benchmark articles), leading us to conclude that the search strategy's comprehensiveness was satisfactory.

### Article screening and study eligibility criteria

#### Screening process

We conducted a two-step screening process to evaluate the eligibility of the articles: first by examining the title and abstract, and then by reviewing the full text. Two separate reviewers independently conducted these screenings using an Excel*®* spreadsheet, and their assessment outcomes were compared for consistency. To ensure consistency, we conducted a calibration exercise (details below) during the screening process. Articles that met the eligibility criteria based on the title and abstract proceeded to the second stage of screening. We conducted a full reading of articles with only a title but without an abstract to determine whether they should be included in the second screening stage. The second stage involved thoroughly reading articles that satisfied the eligibility criteria from the initial screening stage. Any articles that did not meet the criteria at this stage were also excluded. The team held weekly meetings throughout both stages to address any evaluation discrepancies.

To ensure the uniformity and accuracy of inclusion decisions during the screening process, two reviewers assessed consistency by randomly selecting 5% of the total article sample for screening, which included (i) titles and abstracts (n = 175) and (ii) full-text articles (n = 21). Within this sample, the team calculated the consistency rate between reviewers considering the list of articles that met the inclusion criteria for both stages 1 and 2. The results of this consistency check were compared between the reviewers, and any disagreements were thoroughly discussed until the consistency level reached a minimum of 80%. Ultimately, the consistency levels achieved for stages 1 and 2 were 90%.

#### Eligibility criteria

The following criteria were met for the inclusion of each of the articles in this review in (1) title and abstract, and (2) full text:

- Population: We incorporated articles exclusively focused on small-scale populations, including indigenous communities residing in rural areas. Consequently, we excluded articles that: (i) addressed extensive rural properties, such as those engaged in commercial monoculture targeted at commodity markets or agribusiness; (ii) small-scale populations that moved to urban areas, and (iii) those cases in which there was no specification of the target population. Eligible small-scale populations included subsistence farmers or horticulturists, fishers operating small to medium-sized wooden boats, pastoralists, herders or family agropastoralists, and hunter-gatherers or foragers.
- Outcomes: The article tackled the notion of climate change perception, regardless of whether it had been defined explicitly or left to implicit interpretation.
- Study Design Types: we considered empirical studies that relied on primary data and employed a combination of quantitative and/or qualitative data collection methods.

### Study validity assessment

Evaluating the validity and quality of studies is not mandatory in systematic mapping, as per established guidelines [88]. Due to the descriptive nature of this mapping, assessing causal relationships or generalizing findings to certain contexts was deemed unnecessary.

No formal study validity assessment was conducted since the interest and purpose was to describe the variability in usage of the perception concept in the published literature, as a way to obtain an overview of the conceptual similarities and differences in the climate change literature. Therefore, there was no restriction on the choice of eligible articles regarding quality or aspects related to internal or external validity.

### Data coding strategy

To ensure consistency between coders, two team members independently coded a sample comprising 5% of the total number of articles (n = 21). These articles were selected based on their chronological order in the output list, starting with the most recent dates at the top. After coding this sample, the team discussed any discrepancies or uncertainties. In cases of disagreements, a third team member was consulted to reach a resolution.

All eligible articles underwent a dual screening throughout the coding process to ensure consistency. Weekly team meetings were scheduled to tackle any issues and synchronize efforts among reviewers, ensuring consistent coding practices.

During the full-text screening, as previously explained, the team carried out data extraction and coding for the articles that fulfilled the inclusion criteria. For data extraction and coding, the team adhered to a codebook that had been prepared before the mapping process (Additional file 2). The team adopted the following format for data extraction and coding for articles that met the eligibility criteria for full review.

- Bibliographic information: Title, authors, journal, year, DOI.
- Study location: Country.
- Stated origin of the researched population: All non-indigenous, all indigenous, mixed indigenous and non-indigenous, non-specified.
- Subsistence strategy of the investigated population: Forager or hunter-gatherer or fisher-gatherer, horticulturalist, small-scale agriculturalist, small-scale agropastoralist, pastoralist or herder, other.
- Topics addressed in the article: Adaptation, awareness, comparison between individual perceptions with scientific data, traditional knowledge with and without indicators, scientific knowledge, observed changes in the environment, observed changes in livelihood activities, perception determinants or drivers, risk perception, climate change mitigation, resilience, another thematic area.
- Presence or absence of a clearly stated definition of perception: Explicit, implicit, other.
- Section of the text where the definition of perception is found: introduction, methods, results, discussion, conclusion, or elsewhere.
- Explanation of the authors' explicit definition of climate change perception: As provided in the article.
- Identification of the component(s) (“constructs”) included in the definition(s) of climate change perception used, indicating whether perception is assessed in terms of environmental observations, sensory experiences, people's belief in climate change, traditional knowledge, attitudes, and similar aspects.
- Description of how the article presents the perception concept within its results, discussion, or conclusion, irrespective of whether a formal definition of perception was provided. This may include, for instance: Portraying perception as observations of changes, either traditional or scientific knowledge, or an individual's level of concern regarding climate change's impact on livelihood activities, among other aspects (see Additional file 2 for more details).
- Detailing how perception is depicted in the results: Following the authors' description (if explicitly provided).
- Explanation of the phenomenon under investigation in the article: whether it pertains to the physical, biological, human, or another type of phenomenon.
- Explanation of the unit from which data were collected in the methodology: individual, household, both individual and household, community or village, organizations (e.g., associations, NGOs), and any other relevant categories.
- Explanation of the type of data employed in the article for the analysis: primary, secondary, a combination of primary and secondary, and any other applicable categories.
- Identification of the theories referenced in the article as outlined by the authors (when explicitly presented).

After completing the full-text article screening, we compiled an Excel*®* spreadsheet database to document all the studies (Additional file 3). The team collected data exclusively from the main text and did not engage in subsequent communication with the authors to retrieve the missing information.

### Data mapping method

The team compiled and presented the number of articles identified at each stage of this review using a ROSES flow diagram. The information that was extracted was organized into a database in an Excel® spreadsheet format. All explicitly identified definitions of climate change perception have been incorporated in this and are also available in the Additional file 4.

A summary outlining the various constructs and theories presented in the literature has been provided in additional files as described in the results section. We have synthesized the evidence base using bar plots, with a focus on (i) year of publication; (ii) characteristics of the studied population (population’s origin; subsistence activities practiced by the population under study); (iii) definitions of perception; (iv) methodological approaches, and (v) thematic areas. Information about the geographic distribution of the evidence has been gathered and displayed on a map highlighting the countries involved.

## Review findings

### Review descriptive statistics

**Fig. 1:**
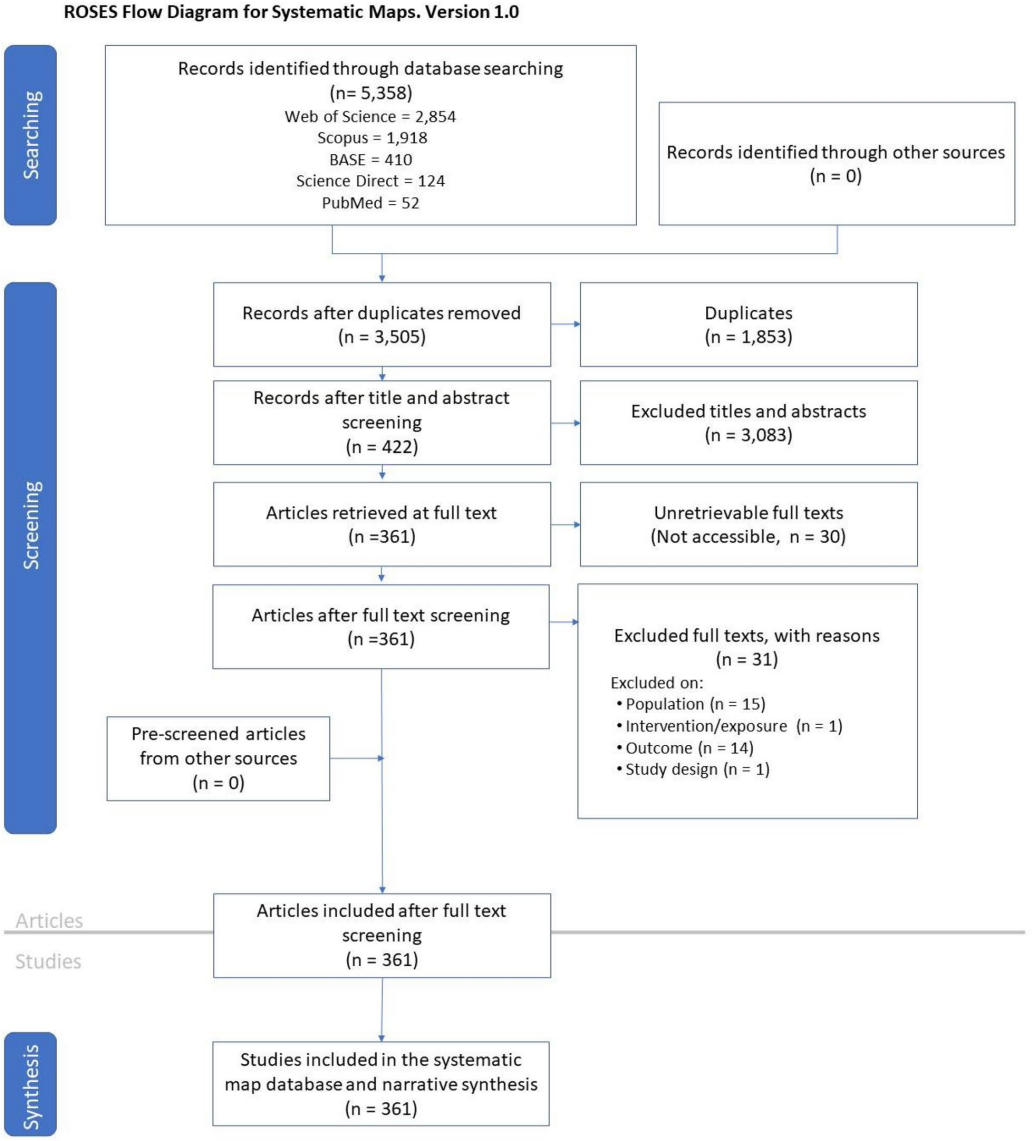
ROSES flow diagram illustrating the literature search and screening process. Source: [89]

In total, 5358 scientific articles were initially identified in the five databases under investigation, and after removing 1853 duplicate articles, there remained a total of in 3,505 articles covering the period between 2018 and 2022. Following the screening of titles and abstracts, 422 articles were initially selected for potential full-text examination. Out of these 422 articles, 30 were inaccessible (due to subscription requirements), and 31 did not meet the eligibility criteria. As a result, a total of 361 articles were included for data extraction during the full-text reading phase. Figure 1 provides a quantitative overview of the search and screening process employed to identify the included articles. For the complete reference list of selected articles, including those that were inaccessible or not selected for inclusion, please refer to Additional file 5. Additionally, for the complete list of articles that were excluded during the title and abstract screening phase along with the reasons for their exclusion, please consult Additional file 6. All data extracted from the articles are provided in Additional file 7.

### Year of publication and geographical location of the fields of study

**Fig. 2:**
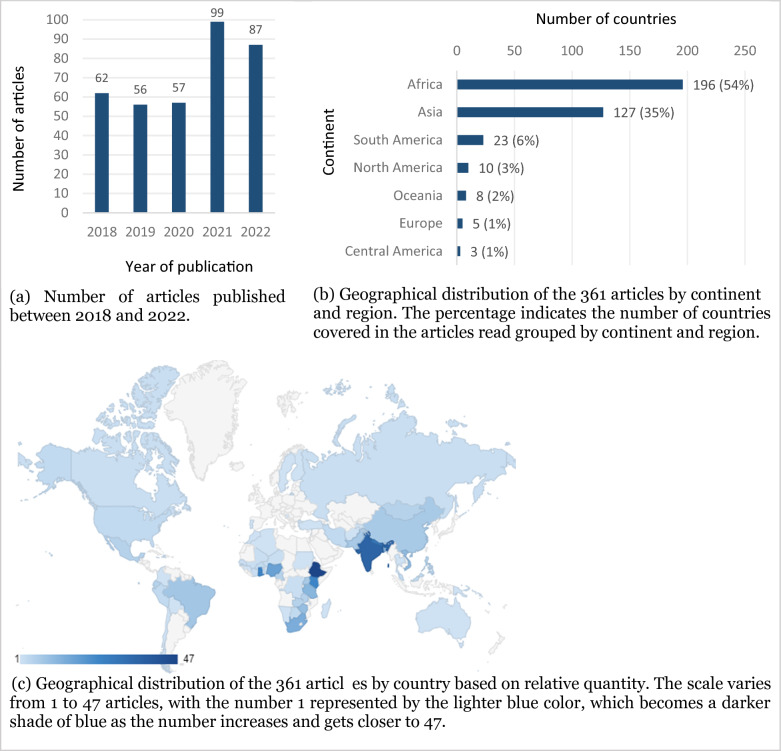
Characteristics of the 361 articles included in the mapping

During the examined period (2018 to 2022), the years that saw the highest number of article publications were 2021 (constituting 27% of total; n = 99), and 2022 (representing 24%; n = 87) (Fig. 2a). This suggests a notable upward trend in the number of publications, indicating a recent surge of in research interest regarding how small-scale populations perceive climate change.

Out of the 361 articles, nearly all (97%, n = 353) focused on a single country, while only five (1.38%) publications encompassed two countries, and three (0.83%) covered three. Continents were represented unevenly, with a substantial proportion of the articles examining small-scale populations in Africa and Asia (Fig. 2b), accounting for 54% and 35%, respectively. Within the African continent, the most frequently studied countries with small-scale populations were Ethiopia (n = 47), Ghana (n = 25), Kenya (n = 24), Nigeria (n = 17), South Africa (n = 14), and Tanzania (n = 12). In the Asian context, this list includes India (n = 36), Nepal (n = 29), Bangladesh (n = 14), Vietnam (n = 11), Pakistan (n = 7), and China (n = 7) (Fig. 2c).

At least two factors may account for the heightened research focus on these countries.

Researchers have dedicated substantial attention to these two continents, as evidenced by their inclusion in 54% and 35% of the 361 articles. This extensive coverage can be attributed to the fact that the populations in these regions are among the world’s poorest and rely heavily on weather conditions for their subsistence activities, rendering them highly susceptible to the impacts of climate change [3, 90]. Both continents are predicted to experience severe changes, including temperature anomalies, rising sea levels, ice sheet melting, and flooding [91]. For instance, Mozambique, Bangladesh, Pakistan, and Nepal were among the top ten countries most affected by extreme weather events between 2000 and 2019 [92]. In Africa, there has already been a temperature increase of by 0.3ºC per decade between 1991 and 2021, resulting in numerous countries grappling with extreme droughts, exemplified by Ethiopia and Kenya [91, 93, 94]. These events may have contributed to a 34% reduction in agricultural productivity in Africa since 1961 [93].

Secondly, the high number of articles may also be indicative of a publication bias, as certain countries have research groups and/or partnerships with foreign institutions that specialize in climate change investigations.

Less studied continents and regions with small-scale populations include: Europa, with Russia^1^ (n = 2), Finland, Sweden, and Bosnia-Herzegovina (all with n = 1); Oceania, with Samoa (n = 2), Vanuatu, Papua New Guinea, Australia, Solomon Islands, and Tonga (all with n = 1); and Central America, with Haiti, Jamaica, and San Vincent and the Grenadines (all with n = 1). In South America, the most researched countries were Brazil (n = 8), Ecuador (n = 5), and Colombia (n = 5); and, in North America, Mexico (n = 5), United States (n = 3), and Canada (n = 2) (Fig. 2c).

Based on the evidence presented, some countries with small-scale populations were not studied. For example, it was expected to find studies in Nicaragua, Costa Rica, Panama, and Guatemala. There are seven indigenous peoples living in Nicaragua and eight in Costa Rica, while 12% of Panama's population and 44% of Guatemala's are autochthonous [4]. Furthermore, we should anticipate a greater volume of research in certain countries, such as Brazil, because the country is home to 305 identified indigenous peoples. The same applies to Bolivia, where 48% of the population is indigenous, albeit many reside in urban areas, and Peru, where four million indigenous people live in varied conditions [4].

There are four plausible reasons to explain the absence of these studies in our sample. The first reason is the challenging research environment that emerged between 2019 and 2022 due to the covid-19 pandemic [95, 96]. Second, certain areas inhabited by indigenous peoples and traditional populations can be challenging to access (e.g., the Wayana indigenous people in French Guyana and Suriname, the Tiriyó people in Suriname [97, 98], as well as populations in Greenland [4]). Third, researchers might avoid specific locations due to risks associated with illicit activities, such as mining near Indigenous Lands (e.g., Guyana) [4], or war and violent conflicts, such as in South Sudan [99], Israel and Palestine [4], Syria, Iraq, Ukraine, Somalia, Chad, Lybia, Yemen, and Saudi Arabia [100]. Fourth, some European countries with small-scale populations did not yield articles (Fig. 2c). We speculate this is possibly due to the lower scientific “appeal” of studying traditional European small-scale populations compared to those in developing countries.

### General characteristics of the investigated populations

**Fig. 3:**
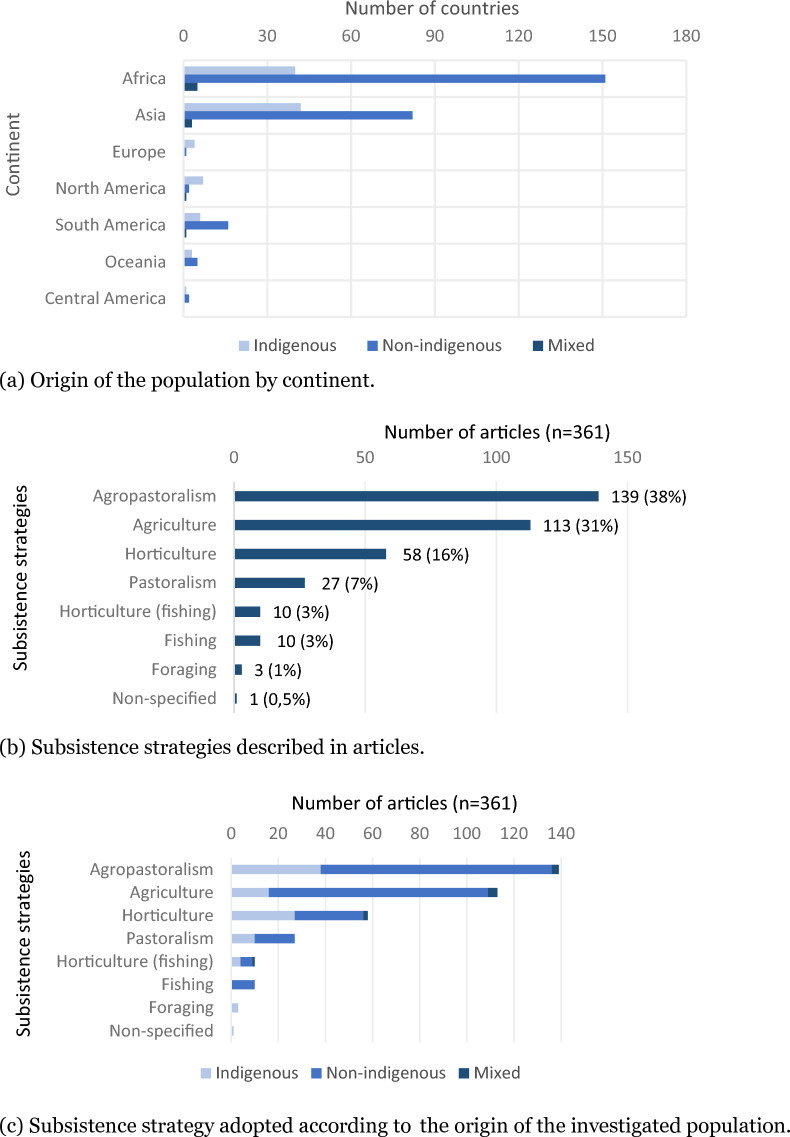
Characteristics of investigated populations: origin and subsistence strategy

Out of the 361 articles, a significant majority (70%, n = 252) focused on non-indigenous small-scale populations residing in rural areas (non-urban, e.g., smallholder, farmer, small-scale fisher/hunter). Approximately one-third (27%, n = 99) addressed indigenous people living in rural areas, referred to in the articles as indigenous people, native people, natives, aboriginal, autochthonous, or ethnic. Only 3% (n = 10) of the articles addressed both indigenous and non-indigenous populations. With exception of the European and American continents, the data indicated that non-indigenous populations were more frequently studied than indigenous ones (Fig. 3a).

The most common subsistence strategies in the studied populations (whether indigenous, non-indigenous, or both) were agropastoralism (a combination of agriculture and pastoralism; 38.5%, n = 139), followed by small-scale agriculture (31%, n = 113), horticulture (16%, n = 58), and pastoralism (7%, n = 27). The least investigated activity was foraging (1%, n = 3) (Fig. 3b), as exemplified by the Twa people, an indigenous group of hunter-gatherers the Democratic Republic of Congo [64]; by the Inuit in Canada [101], and five other ethnicities in the United States (Quinault, Salish Kootenai, Siletz, Shoshone, Shivwits Band of Paiutes) [102].

When focusing on indigenous populations, the same pattern emerged, with the exception of horticulture, which was more commonly practiced (7%, n = 27) than small-scale agriculture (4%, n = 16), consistent with the traditional activities of indigenous communities worldwide [4]. Among non-indigenous populations, small-scale agriculture (26%, n = 93) was less common than agropastoralism (27%, n = 98). These findings align with expectations, as agriculture and pastoralism are relied upon by 78% of the world’s poorest population, who reside in rural areas [103] (Fig. 3c).

### Thematic areas

All 361 articles on climate change perception covered multiple topics. As anticipated, 97% of the articles (n = 349) delved into environmental changes (e.g., increase in drought) and their resulting impacts. Similarly, 94% of the articles explored changes in the livelihoods of small-scale communities, specifically their ability to satisfy basic survival needs such as water, food, shelter, and clothing (n = 340). The majority (74% of the articles; n = 267) also examined adaptation to these changes. The frequently addressed themes included gender issues (2%, n = 7), health and food security (3%, n = 10), vulnerability (7%, n = 26), mitigation of the effects of changes (12%, n = 44), and risk perception (13%, n = 46). For a comprehensive table with reference examples for the thematic areas, please refer to Additional file 8.

**Fig. 4:**
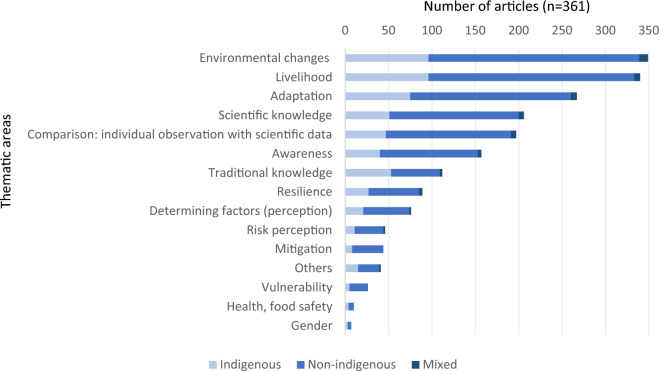
Thematic area by origin of the investigated population

The topic of traditional knowledge was slightly more frequently addressed among non-indigenous populations (n = 56, 50%) than among indigenous populations (n = 53 or 47%) (Fig. 4). Of these (n = 112), 39% (44 articles) focused on physical and/or biological indicators derived from environmental observations. For instance, studies among indigenous peoples (e.g., in Bolivia [104], South Africa [105], Namibia [106], and Vietnam [107]), and among non-indigenous people, for example, in Zimbabwe [108], Mexico [109], and Nigeria [110] presented traditional knowledge related to various climatological predictors. These included precipitation, storms, droughts, unusually hot or cold years, and floods, assessed through atmospheric (clouds, wind), astronomical cues (moon phases, star positions), and biological indicators (flora and fauna).

### Methods used in the articles

More than half of the publications (61%, n = 222) utilized both primary and secondary data in their analyses. In contrast, the remaining publications (39%, n = 139) relied solely on primary information, which refers to data collected directly from the studied population. Secondary data sources encompassed various types, including data from meteorological stations (e.g., [58, 70, 72, 111–113]), satellite-based precipitation estimates (e.g., [114]), official government data (e.g., [115]), census information (e.g., [116]), or literature reviews (e.g., [71, 113]). However, recall that no publications exclusively based on secondary data were identified, as one at the inclusion criteria specified the presence of primary data.

**Fig. 5:**
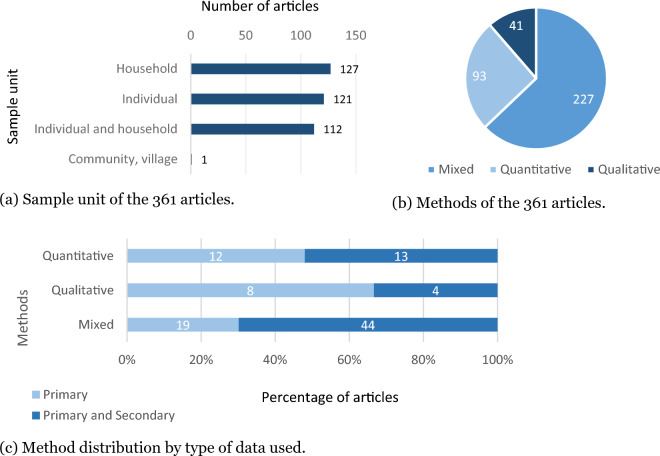
Proportion and number of articles (above bars) with different methodological approaches (total number = 361)

The sampling unit most commonly employed in the articles was the household (35%, n = 127). However, a nearly equal proportion of articles focused on the individual (33%, n = 121), with the remainder of them encompassing both. (Fig. 5a). There were exceptions, such as Salvadeo's study [60], which examined the community as the sample unit.

Regarding the methodology used, mixed-method approaches (combining qualitative and quantitative methods) were employed in a larger proportion of the publications (63%; n = 227), while about one-third utilized exclusively quantitative methods (26%; n = 93) (Fig. 5b). When analyzing the combination of methodological approaches (quantitative, qualitative, mixed) and data types used (primary, primary, and secondary), it becomes evident (Fig. 5c), that the most common approach (n = 160) involves employing mixed methods and incorporating both primary and secondary data.

### Theoretical approach of the articles

Out of the 361 articles, 43 (22%) incorporated 18 different theories. A small proportion of the reviewed articles explicitly mentioned at least one theory (10% of all articles, n = 36), while 2% (n = 7) presented multiple theories [9, 66, 69, 117–120]. Additional details on how these theories were utilized or cited in the articles can be found in Additional file 9.

The large majority of articles (88%) do not appear to be based on theories, at least explicitly mentioned, indicating a limited theoretical foundation in this research area. Furthermore, even in studies with theoretical foundations, the theory was often not evaluated in the article’s results, except for some theoretical frameworks like the Capital Approach Framework [121, 122]*.* Other theories were only briefly referenced in the articles (e.g., Integral Theory, Norgaard's Theory of Denial, Grounded Theory) [108, 117, 118, 123]. Additionally, very few articles (4%, n = 15) combined at least one theory with a definition of perception.

Given the limited focus on theoretical approaches, it can be inferred that there is no consensus on which theories should serve as the foundation for studying climate change perceptions among smallholders. The majority of theories adopted come from Psychology (35%) and Economics (23%), with some being multidisciplinary (12%). The most commonly addressed approach is Cognitive biases, which were discussed in 16 articles. This is followed by Theories of Rational Choice, particularly the Expected Utility Theory, which appeared in eight articles. These articles explored cognitive biases as psychological barriers to accurate climate perceptions [9, 69, 124, 125] or as factors influencing individual decision-making [30, 55, 118].

Additional theories were utilized to elucidate climate change perception and risk perception, including Cultural Theory [126], Value-Believe-Norm Theory [127, 128], the Perceptual Geography Approach [129], the Theory of Planned Behavior [130, 131], and the Model of Private Proactive Adaptation to Climate Change [66, 132]. The Expected Utility Theory was applied to gain insight into how individuals make choices regarding adaptation strategies to maximize their utility, considering factors like higher profit or reduced impact of climate change [133–136].

In response to climate risk, the behavior of individuals was explored through the Prospect Theory [69], and this behavior could influence the choice of adaptation strategy, as elucidated by the Protection Motivation Theory [54, 137, 138]. Other theories also delved into adaptation, such as the Climate Change Response Model [70], which examined the connection between vulnerability, perception, and adaptation strategies. Lastly, the Capital Theory (or Capital Approach Framework) examined the relationship between perception and adaptive assets or adaptation strategies [122, 139].

In summary, the literature in the area is seldom underpinned by theoretical frameworks. When articles incorporate theory, the explanations for perception are frequently rooted in Psychology and rationality, except for cognitive biases and the Prospect Theory, which provide non-rational explanations.

### Explicit definition of perception and perception constructs

**Fig. 6:**
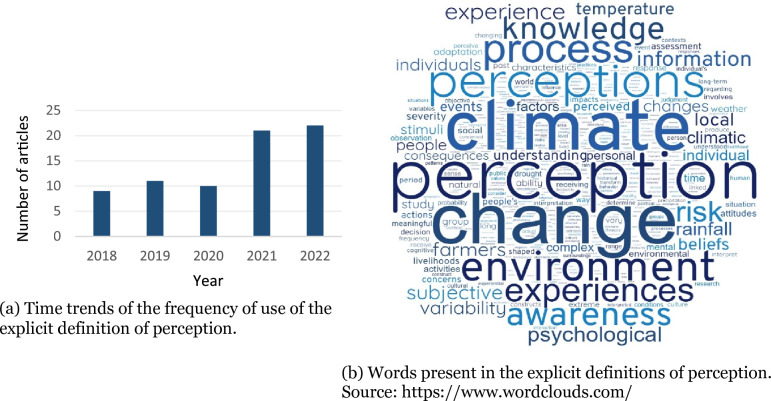
Information about the definitions of perception

Out of the 361 articles, only 20% (n = 73) included an explicit definition of the concept of perception. A smaller percentage (4%; n = 15) presented a theoretical definition throughout the article (see Additional file 9), rooted in specific theories of the Human or Social Sciences. As anticipated, when comparing the years 2018 to 2022, there was an increase from 12% (n = 9) to 30% (n = 22) in the use of explicit concepts in published articles (Fig. 6a). Among the articles that provided an explicit definition, the vast majority presented the concept only in the introduction (80%, n = 58), with a smaller number mentioning it exclusively in the Methods section (8%, n = 6), the Results (8%, n = 6), or the Discussion (4%, n = 3).

The explicit definitions identified in the articles were categorized into seven groups, with inclusion criteria detailed in Table 1. These groups reflect the primary context of the explicit definition utilized. Each definition was assigned to a group based on its main characteristic, even if specific definitions could potentially fit into more than one group. All explicit definitions are presented in Table 2.

**Table 1:** Description of the criteria for inclusion in one of the seven perception groups

Groups of definitions of perception	Criteria for inclusion in the group
Risk perception	Perception is defined by terms such as risk perception(s) or perception of risk
Awareness	Perception is defined through awareness by using the following words: awareness, climate change awareness, physiological or psychological awareness
Perception according to psychological constructs, environmental and/or sensory stimuli	Perception is defined according to the Cognitive Psychology approach, mentioning psychological constructs, sensory perception (seeing, hearing, experiencing, interpreting, organizing sensations, and others), cognitive processes, or information and stimuli received
Prior experience	Perception is defined according to the individual's prior experience with climate change or with the effects caused by extreme weather events. It may be described as personal experience, lived experience, interpretation of environmental experiences, or social experience
Observation of climate variables	Perception is defined through the observation of climate change, climate variability, weather extremes, or raw data processing
Individuals' beliefs and interpretation of the environment	Perception is defined as the interpretation of weather events, based on experiences, beliefs, or understanding
Uncertainty and threat	Perception is defined based on the uncertainty or threat of a weather event

**Table 2:** Explicit definitions of the concept of perception divided into categories (n = 73 articles)

Definition of perception	References
1. Risk perception	
*"Based on the IPCC’s definition of “risk perception,” we consider “perceptions of change” as “the subjective judgment that people make about the characteristics and severity of changes.”"*	[140]
*“Risk perceptions are beliefs about potential harm or the possibility of a loss. This is a subjective judgment that people make about the characteristics and severity of a risk. In the study, risk perception was considered to be a subjective judgment made by farmers regarding the characteristics and severity of the risks brought about by climate change."*	[128]
*"(…) Risk perception (i.e., person’s subjective judgment or assessment of risk)"*	[127]
*"(…) the perception of risk is a subjective judgment of the likelihood of a respective event such as flood, drought, cyclone, *etc*. and stakeholders’ subsequent awareness of its level of damage. (…) The study defines hailstorm risk perceptions as concerns shown by farmers regarding previous, present, and future incidents of negative impacts on crop production and agricultural activities due to the occurrence of hailstorms"*	[141]
*"Climate change risk perception is a multitask procedure that depends on different factors including socio-economic, demographic, political, and cultural activities. Overall, personal understanding leads to a pivotal role in identifying farmers’ perceived climate-related risks"*	[135]
*"(…) perception of CC is a personal assessment that comprises an individual’s understanding, which in turn motivates actions with respect to CC incidence and severity. Thus, an individual must perceive CC before responding to it, and this perception needs to be linked with actual CC for effective adaptation measures". (…) The perception of risk is a mental construct and personal perception may vary among individuals"*	[142]
*"Risk perceptions refer to a decision maker’s assessment of the risk inherent in a situation. They are important determinants of decision maker behavior as studies have shown they can influence the assessment of uncertainty and distort one’s judgments, knowledge, and the ability to perform under risky conditions. They are generally measured by asking about the perceived “seriousness,” “concern,” and/or “worry” of a situation". (…) In the psychology literature, perception refers to the process of receiving information and stimuli from one’s surroundings and converting them into psychological responses. The perception of risk is, therefore, a mental construct that distinguishes between the existence of objective real-world threats and the subjective evaluation of those threats"*	[69]
*"Risk perception is a mental construct and farmers’ climate change risk perceptions are unique in a sense that it allows for a differentiation between the actual real-world hazards, for instance, climate change, and intuitive evaluation of those dangers"*	[143]
*"Risk perception involves the “subjective assessment of the probability of a specified type of accident (or event) happening and how concerned we are with the consequences”"*	[119]
*"(…) risk perception is the subjective assessment of the probability of a natural hazard occurring and the consequences of hazards activities (severity)"*	[137]
*"Threat appraisal, also known as risk perception, is the primary cognitive process assessing how an individual is threatened by a specific known risk consisting of perceived probability and perceived severity (the consequences)."*	[54]
2. Awareness	
*"Climatic perception is defined as a state of opinions and/or awareness toward the changes in climate variables."*	[66]
*"The degree of awareness for climatic variabilities is time and space context-specific, which varies according to local communities’ own experiences with their ecosystem. The preliminary knowledge of climate change comes from the direct observation of the environment and its physical consequences."*	[144]
*"Perception is the process of receiving information from the ambient environment and transforming it into physiological awareness for taking adaptation and mitigation strategies towards adverse impacts of climate change in the agroecological system. However, this process could vary with the individual’s past experiences, observations, and present attitudes, needs, and social circumstances and also depending on one´s livelihood, literacy, and settlement."*	[145]
*"According to the encyclopedia of qualitative research methods, perception is like a set of lenses through which an individual views reality. In this study, the perception of climate change was assessed through the frequency of “awareness” or “knowledge” of climate change and how the interviewees observe the changes in temperature, rainfall, and spatial–temporal distribution of rain."*	[146]
*"Climate change awareness involves creating knowledge, understanding and values, attitude, skills, and abilities among individuals and social groups towards the issues of climate change for attaining a better quality environment. (…) In this study, awareness of climate change including (i) conceptual awareness; (ii) experiential awareness; (iii) engagement awareness, and (iv) adaptation awareness. Conceptual awareness regards an individual’s knowledge on the causes of climate change; their impacts and the necessity for a response. Experiential awareness concerns experiences and knowledge of long-term changes in climatic conditions and associated impacts on the availability of resources and livelihoods. Engagement awareness is about the frequency with which an individual talks or hears about climate change, while adaptation awareness refers to knowledge on climate forecasting, adaptation techniques and climate response policies."*	[147]
*"Farmers’ perception of climate change was considered as an aggregated awareness about the trend of the following four climatic parameters (rainfall, temperature, number of rainy days and frequency of dry spells) generated from the historical climate records of the research area."*	[148]
*"Climate change perceptions are the process of receiving information from the environment and transform it into psychological awareness."*	[149]
*"Farmers’ perception of climate change refers to an aggregated awareness of the trend in the climatic parameters such as rainfall, temperature, drought and onset and end of the rainy season."*	[150]
*"A plethora of scholars define climate change perceptions as awareness of change in climatic conditions and their impacts on people’s livelihoods."*	[108]
3. Perception according to psychological constructs, and environmental and sensory stimuli	
*"Recent literature revealed that climate change perception is a challenging process that involves psychological concepts, such as attitudes, beliefs, and concerns on how climate change is happening. Perception, in this case, refers to people’s understanding of the reality and causes of climate change, its consequences, and the factors that determine the decision to apply appropriate measures.”*	[131]
*"Essentially, climate change and extreme events perception are complex processes that encompass a range of psychological constructs, such as knowledge, beliefs, attitudes, and concerns about whether and how the climate is changing."*	[151]
*"Perceptions about CC are “a complex process that encompasses a range of psychological constructs such as knowledge, beliefs, attitudes, and concerns about if and how the climate is changing."*	[152]
*"Perception refers to the process in which people receive information and stimuli from their environment and transform them into conscious psychological actions."*	[49]
*"It is fundamental to consider that the perception of climate change is a complex process that encompasses a variety of psychological constructs, such as the knowledge, beliefs, attitudes, and concerns about whether and how the climate is changing. Perception is influenced and shaped by, among other things, the characteristics of individuals, their experiences, the information they receive, and the cultural and geographic contexts in which they live."*	[122]
*"Environmental perception is the response of the senses to environmental stimuli (sensory perception) and the mental activity resulting from the relationship with the environment (cognitive perception)."*	[153]
*"Farmers’ perception, which is a cognitive driving force."*	[121]
*"Farmers’ perception of climate variability is a complex process that includes a range of psychological constructs such as knowledge, beliefs, attitudes, and practices related to how the local climate has varied. Farmers’ perception of climate variability is shaped by farm household characteristics, historical experiences with local climates especially the impact of climatic changes on agriculture productivity, the knowledge that they receive, socio-cultural and geographic contexts where farmers cultivate their fields. In this study, farmers’ perception of climate variability was defined by their experiences during the decade which preceded the survey in Tharaka-Nithi County (2007–2017) regarding seven climatic characteristics and several consequences that they had experienced as a result of climate variability. These indicators included change in temperature, change in rainfall amounts, change in rainfall onset and rainfall cessation dates, change in length of the cropping season, and changes in flooding and drought frequency. The consequences of climate variability that shaped farmers’ perceptions in Tharaka-Nithi County included changes in soil fertility and soil erosion risks, changes in agricultural productivity, and changes in natural and planted forest cover."*	[41]
*"Perception has been defined as the process by which organisms interpret and organize sensation to produce a meaningful experience of the world; and that a person’s perceptions are based on experiences with natural and other environmental factors that vary in the extent to which such perceptions are enabled. Farmers’ perception of climate variability refers to their lived experiences about fluctuations in weather patterns (especially rainfall, temperature, and drought) and how that affected their livelihoods."*	[57]
*"Perception is defined as a process of receiving information and stimuli from our surroundings and converting those into psychological responses. However, individual perception differs with time and situation and particularly, perception of climate change is a difficult idea for the farmers."*	[154]
*“Perception is a process in which stimulus or information is received and transformed to generate a psychological awareness. This stimulus is formulated based on cultural background, prior experience, and socioeconomic factors.”*	[155]
*"In this study, perceptions about climate variability and other stressors were defined as an individual’s ability to see, hear and experience (over the period 2000–2015) any one or combination of stressors caused by climatic phenomena alone and/or ecological, socio-economic and political factors affecting activities vital to the farmers’ subsistence.”*	[156]
*“(…) farmers’ perception refers to short-term experience relying on memories.”*	[157]
*"Perception is the process of receiving external stimuli and converting them into psychological responses based on past events and the present situation."*	[158]
*"Perception is the first cognitive process through which the individual obtains information from the environment and allows the subject to form a representation of reality."*	[159]
*"(…) public perception, defined as the process by which the public interprets and organizes sensation to produce a meaningful experience of the world (…)"*	[160]
*"Van den Ban and Hawkins (2000) define perception as the process by which we receive information or stimuli from our environment and transform it into psychological awareness to produce meaningful experiences of the world."*	[42]
*"As pointed out by an anonymous reviewer, ‘perception’ and ‘understanding’ are sometimes synonymous. Yet, the Oxford Living Dictionary contains two distinct definitions of the word perception – ‘The ability to see, hear, or become aware of something through the senses,’ and ‘The way in which something is regarded, understood, or interpreted.’"*	[161]
*"Perception here follows the definition of Ndamani and Watanabe (2015) as the process by which organisms (humans) interpret and organize sensations to produce a meaningful experience of the world."*	[162]
*"The perception framework is hinged around psychology, which is study of behavior and mental processes. (…) perceptions are subjective and comprise a wide range of things which are contextual, value-laden and dynamic. For example, a definition of a similar event might be different within a group of individuals in with experience, i.e., how individuals react to situations. This is so because perception is a function of the actions displayed thereafter."*	[163]
*"Perception of the environment describes how a person perceives the environment through the brain´s and their senses’ ability to process and store information. The perceptual process is highly complex, but broken down it consists of six steps: the presence of objects, observation, selection, organization, interpretation, and response. The selection, organization, and interpretation is personalized and driven by internal and external factors. For example, the motivation, personality, or experience of an individual plays a role in how they perceive their surroundings, but also a continued repetition of being exposed to an object or a situation can alter their personal perception."*	[164]
*"Perception to climate variability can be associated with both social-cultural construction and psychological dimensions. From a social cultural dimension perception it is systematically determined by how people who share a common culture interpret a phenomenon that affects their livelihoods and way of life. Psychologically, perceptions may vary from person to person or from group to group. However group differences in perceptions are often larger to result to predictive differences in perception between those groups. Such group dynamics may be due to gender, culture, livelihood activities, geographical locations, income age and level of education. (…) perception may be shaped by social variables that include culture, political and psychological factors since they all determine how people interact with the natural environment, including their livelihood practices."*	[165]
4. Prior experience	
*"The local perspective comprises perceptions of changing weather patterns, related traditional ecological knowledge (TEK), and experiences of an extreme precipitation event, which all influence local decision making in natural resource management matters."*	[104]
*“In this sense, public perception of climate change can be interpreted in a temporal context. (…) In this sense, people’s experiences of weather events over time form their perceptions. Therefore, public perception of climate change may relate to past experiences, current phenomena, and predictions of what will happen and how it will affect their lives.”*	[58]
*"Perception is described as the process of creating experiential feelings in the real world and highlighting an individualʼs ability to take advantage of his experience of nature and natural variables."*	[40]
*"The theoretical context of climate change perception is built on observation, personal experience and information received from the surroundings/neighborhood over a period of time."*	[166]
*"Perceptual geography is characterized by a common idea that experience affects perception, which leads to the conclusion that perceptions vary because individuals’ life experiences differ. Perceptions are understood as points of contact between people and their environment and as a basis for spatial reasoning and decision making. Perception is the process that encodes the objective environment as a subjective one, with the subjective environment and past experiences influencing our behavior and actions". (…) Perceptions also carry culture, and local and traditional knowledge. This means knowledge and practices, developed during centuries and handed down from generation to generation."*	[129]
*"(…) perception is mediated by and modified through interaction with the environment, historical background, and personal or lived experiences. In the context of climate change, perception is often studied as the process of acquiring information about one’s environment and how it enhances climate awareness."*	[167]
*"Personal perception is what individuals perceive of the local climate instability, climate change and reactions, based on personal experience and values."*	[168]
*"Perception is a cognitive process through which humans interpret experiences of the environment and in turn generate response strategies. Schlüter *et al*. (2017) highlights that in various behavioral models, perception is the initial receptor stage, i.e., “what comes in” and behavior is the final outcome, i.e., “what goes out.”"*	[169]
*"Local people’s perception of rainfall behavior is an idiosyncratic manifestation of their experience and various environmental aspects."*	[170]
*"Perceptions are complex and dynamic processes that are tied to social experiences and constitute a bridge between lived contexts and the environment."*	[171]
*"Slegers (2008) and Ejembi and Alfa (2012) add that human perceptions of environmental changes are informed by experiences of how the changes influence people’s livelihoods."*	[172]
5. Observation of climate variables	
*"(…) perceptions of climate change were defined as people’s perspectives on local-scale changes in the state of weather-related factors, such as increased temperature, prolonged droughts, sea level rise, changes in precipitation patterns and large floods in a given area over the last decade, which if they persist over long periods of time become indications of climate change."*	[173]
*"Smallholder farmers perceptions of changes in both temperature and rainfall revealed that perceptions are made based on local environment and are not linked to an understanding of climate change and variability in the national or global contexts."*	[68]
*"The perceptions are usually understood by examining how climate variability (e.g., temperature and precipitation) and climate hazards (e.g.,, drought, storms, and floods) impact Indigenous livelihoods and wellbeing."*	[174]
*"This paper used perception as a way of everyday and long-term interaction with the farmers to process raw data into actual trends."*	[105]
*"CCP (climate change perception) can be defined as the stage at which a household perceives changes in climatic conditions."*	[175]
*"Farmers' perceptions consider farmers' observation of changes in climate and climatic events over a long period. Furthermore, perception refers to the practical knowledge rising from experience and concrete situations; and perception is also linked to local knowledge (…) In this paper, farmers' perception of climate change is defined as the farmers' perception of changes in the climate based on observation and individual experience in relation to the increase, decrease or no change in rainfall, temperature, and extreme weather events over a long period of time."*	[176]
*"Furthermore, perception is the practical knowledge arising from experiences and concrete situations; and perception is also linked to local knowledge. In this paper, farmers’ perceptions of climate change are defined as the farmers’ understanding of climate change based on observations and individual experiences in relation to the increase, decrease or status quo in rainfall, temperature and extreme weather events over a long period of time."*	[177]
*"In this study, perception is defined as the way in which climate change and variability is regarded, understood, or interpreted by local people. Perception is of particular interest because of its ability to enhance solutions for risky climate events or otherwise."*	[178]
*"We differentiate between perceptions of climate change in short and long term. Short-term perception is defined as the perception of extreme weather events in the past year, whereas long-term perceptions are perceived changes in temperature and rainfall over the last 20 years."*	[53]
*"Climate perception is a process by which individuals sense and realize changes in climate-related stimuli, where stimuli include changes in climate variables and extremes."*	[179]
*"Perception is the way of processing raw data that a person receives through his/her daily and long-term interaction with immediate environment into meaningful pattern."*	[180]
6. Beliefs and interpretation of the environment	
*"Perception in this content entails the approaches through which the people understand their environment and so can utilize the environmental resources and acquire the capability to adapt to the stimuli that may arise from their interactions."*	[181]
*"Perception research, according to Kamau, (2010) thus attempts to understand the complex interrelationships between man and the biosphere since man’s actions and decisions concerning the environment are based on objective as well as subjective factors. Perception research is therefore concerned with how individuals or groups perceive their environment and how they react to changes in the environment. Perception is also about the beliefs an individual or a group have about an issue. Perception therefore forms the basis upon which knowledge is derived. (…) Perception therefore helps to determine the social or mental picture of climate change that individuals have and their beliefs about the effects of climate change."*	[39]
*"Climate change perceptions include the individuals’ views and interpretations of the climate issue based on beliefs, experiences, and understanding."*	[182]
*"Perception of climate variability is complex, and involves the opinions, beliefs, values and rules people have regarding climate change, which determine the orientation of their actions, in other words, whether they are positive or negative as regards adaptation."*	[183]
*"Perception refers to the process concerned with the acquisition and interpretation of information from one’s environment."*	[184]
*"We defined perceptions as the views and interpretations of the climate change issues based on beliefs, experiences and understanding."*	[185]
*"Human perception of the environment shapes and is shaped by human knowledge of the environment, and involves interpretation of events or information; therefore, any landscape consists of two basic elements, the biophysical components of an area affected by human activities and analyzed through “objective” analysis, and the perception and the value assigned to the environment by people, evaluated through “subjective” analysis."*	[186]
7. Uncertainty, threat	
*"To farmers, climate change is not perceived in terms of major disasters, but rather as increased uncertainty, such as shifts in onset of rain at planting or end of rain at harvest."*	[31]
*"Perceived probability and perceived severity of a hazard are defined as a person’s expectancy of being exposed to threats and how harmful the consequences of the threat would be if it were to actually occur, respectively."*	[132]

CC: Climate Change. References that were present in the definitions have been removed. For more details on the page where the definition can be found, see Additional file 4

Certain words appeared more frequently in the definitions of perception, including terms like awareness, knowledge, experience(s), risk, process, environment, information, psychological, beliefs, subjective, personal, understanding, and stimuli (Fig. 6b). This observation indicates that climate change perception definitions amalgamate elements from Cognitive Psychology (e.g., stimulus, awareness) with other constructs, such as experience, beliefs, knowledge, and understanding.

**Fig. 7:**
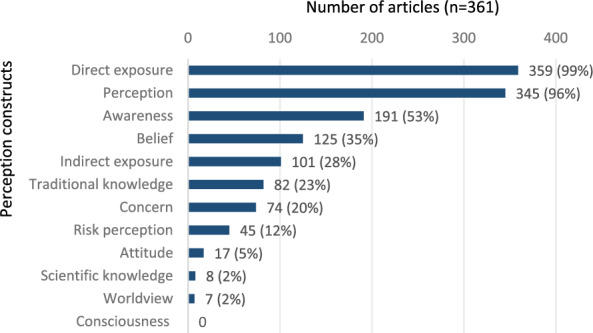
Perception constructs adopted in the 361 articles

All 361 articles included at least one perception construct. The most frequently utilized constructs for defining the concept of perception, either explicitly or implicitly, were direct exposure (99%, n = 359) and perception based on sensory stimuli (96%, n = 345) (Fig. 7). Interestingly, none of the sample articles incorporated the construct “consciousness.” In Cognitive Psychology, consciousness is defined as the “state of wakefulness, the ability to control behavior and be aware of surroundings and mental experiences” [187]. It encompasses various psychological constructs, such as attitudes, knowledge, beliefs, values, and actions (behavior) [188, 189]. Environmental consciousness, or environmental concern, is considered a synonym of consciousness in the context of environmental issues [168]. It can indeed lead to engaging in pro-environmental behaviors as a response to climate change. This means that individuals with a heightened environmental consciousness are more likely to take action to mitigate, minimize, or avoid the adverse impacts of climate change. However, it's worth noting that this construct was not incorporated into the articles, possibly because climate change perception primarily focuses on understanding individuals' awareness and comprehension of climate change rather than directly addressing their subsequent behaviors or pro-environmental actions, which are typically studied in the context of the adoption of adaptive strategies.

In the context of climate change literature, it is evident that the constructs implicitly defining the concept of perception extend beyond the purview of bottom-up or top-down theories within Cognitive Psychology. This departure is due to the fact that, in studies of climate change perception, the literature on perception constructs encompasses more than merely sensory stimuli, which is what we might anticipated with using bottom-up theories. Furthermore, the literature does not exclusively revolve around prior experiences (both direct and indirect exposure) or knowledge (whether scientific or traditional), which are typically associated with top-down theories.

Instead, the concept of perception in climate change literature encompasses a broader array of constructs. These include individual beliefs, feelings of concern, risk perception, attitudes, diverse worldviews, and even an individual's awareness of climate change, as depicted in Fig. 7.

This underscores the interdisciplinary nature of research into climate change perceptions. For additional details about these constructs, please refer to Additional file 10. Additionally, you can explore the distribution of these constructs by geographical region in Additional file 11.

Among the 361 articles, each one included at least two perception constructs. However, not every article that contained one construct necessarily contained another specific construct. For example, articles featuring the construct of “perception” did not necessarily include the construct of “awareness”—the same pattern applied to other constructs. Nevertheless, there were two exceptions to this trend.

Firstly, the construct of “direct exposure” consistently appeared in combination with various other constructs such as “concern”, “indirect exposure”, “risk perception”, “attitude”, and “worldview”.

This result leads us to consider four conjectures: (i) Individuals who perceive climate change as a concern probably have experienced and been exposed to its effects. (ii) Climate change perception could arise from personal experiences with the changes or from experiences acquired from other people or the media. (iii) Risk perception, i.e. the subjective judgment of climate risk, is likely related to having experienced a weather change; (iv) Attitudes and worldviews could be influenced by experience and exposure to a weather change.

Secondly, in the results, articles that addressed perception through scientific knowledge also addressed perception according to sensory stimuli. According to Cognitive Psychology, this implies a relationship between having scientific knowledge and perceiving climate change.

In general, the vast majority of articles (86%, n = 312) described perception (in the results, discussion, or conclusion of the articles) as the observation of climate change in specific environmental aspects through sensory stimuli and awareness of the effects of climate change.

The environmental aspects mentioned include descriptions of the observation of extreme events or climate variability anomalies. These descriptions can be individual reports of changes frequently or occasionally observed, including increases or reductions in factors such as precipitation, drought, temperature, and/or biophysical indicators, as well as others related to changes, such as in the flowering of plants. In these articles, the term "extreme" events or "anomalies" often refers to phenomena such as storms, landslides, tornadoes, wildfires, prolonged droughts, heatwaves and cold waves, frosts, lightning, cyclones, hurricanes, windstorms, or floods. For instance, agropastoralists [111] and pastoralists [190] in Nepal have reported a significant increase in summer temperatures over the last few decades, accompanied by a decrease in the amount of snow. Farmers have also documented unexpected occurrences like rains, droughts, floods, and violent winds [191].

Other studies have documented changes in plants, plant parts, and animals. For example, indigenous people in Zimbabwe have noted a decrease in the availability of wild berries and an increase in the insect population due to climate change. Additionally, farmers in Uganda have reported an increase in pests and a reduction in the number of native trees, besides the occurrence of extreme events, such as increased temperatures and droughts, shorter rainy seasons, and higher frequency and intensity of extreme weather events [192].

A small percentage of the articles (5%, n = 18) described climate perception, including what climate change is, its effects, and causes, based on people's traditional knowledge, sometimes incorporating weather predictors and indicators. For example, one study demonstrated that indigenous people in Bolivia rely on atmospheric, astronomic, flora, and fauna indicators when observing the environment to predict weather phenomena [104]. Among farmers in Ethiopia, climate change perception primarily relies on traditional knowledge [193]. Another study reported varying levels of traditional knowledge about climate change among indigenous people in South Africa [105]. These locals have been using indicators based on flora and fauna to make meteorological predictions and inform decisions related to planting. In Ghana, farmers utilize traditional meteorological knowledge, including flora and fauna indicators, to predict the rainy season and anticipate the occurrences of lightning [194].

An even smaller percentage (2%, n = 8) of articles contain descriptions of climate perception or climate variability perception that rely on information provided by people from outside the community and/or by the media, such as radio, television, newspapers, and magazines. Among these eight articles [42, 60, 69, 175, 195–198], agropastoralists in Burkina Faso used radios, casual discussions, and input from people outside the community to gather information about climate change [195]. A study with farmers in South Africa demonstrated that participating in a farmers' association and listening to the radio heightened their perception of climate change [175]. Indigenous people in Niger acquired information about climate change through local radio, television, farmers' associations, and phones [196]. In Bangladesh, indigenous people gathered information from television, newspapers, non-governmental organizations, and researchers [42].

The same percentage of articles described perception as the degree of concern individuals have about climate change's effects on their subsistence activities. For example, in one study by Budhathoki et al. [137], farmers in Nepal were less concerned about cold periods and heatwaves, and more concerned about floods in agriculture. Even so, they reported that all these events, together with droughts, were the main climate risks. Lone et al. [199] documented the apprehensions of Indian farmers regarding the impacts of climate change on agriculture. In Colombia, Eitzinger et al. [200] demonstrated that older agropastoralists expressed greater concern about climate change compared to their younger counterparts. Nevertheless, in Zimbabwe, agropastoralists were more concerned about the effects of climate change on agriculture and livestock production [201].

One article measured the concept of perception based on individuals' scientific knowledge of climate change. This was accomplished by employing a knowledge test created through consultation with various sources and bibliographic databases, as well as with experts in climate change [202].

Lastly, 4% of the articles (n = 14) did not present any description of respondents' perception of climate change in their results (e.g., [54, 122, 126, 130, 203–211]). Although these articles aimed to assess the perception of climate change, their results went in different directions. For example, based on Capital Theory, Torres et al. [122] assessed climate change perceptions by dividing respondents into five quintiles without describing each group's perceptions. Using exploratory factor analysis and regression models to analyze the data, Alam et al. [211] also did not present respondents’ perceptions in the results. Another article, by Budhathoki et al. [54], only referred to adaptation strategies.

Ng’ombe et al. [209] presented an average level of awareness of climate change and focused on perception determinants. Sarker et al. [210] calculated a climate change perception index to describe perception. Salite [208] focused on beliefs about droughts and the reasons behind these beliefs. Quandt [204] addressed resilience perception in livelihoods and the correlation between drought perception and other variables, such as ethnic group. Makondo et al. [205] focused on changes in dietary habits, migration, taboos, spirituality, and ecosystem services to examine traditional knowledge and awareness of climate change and related environmental risks. Lemahieu et al. [207] addressed changes in the environment. Ambrosio-Albala et al. [126] used the Q methodology to explore and compare worldviews according to Cultural Theory. Lastly, Khanal et al. [206] analyzed determinants and impacts on adaptation strategies.

For the complete description of all excerpts extracted from the results of the 361 articles, as provided by authors (when explicit), see Additional file 12.

### Observed phenomena

Perception through the observation of physical phenomena was reported in 96% of articles (n = 346). These articles described the changes individuals observed (or did not observe), such as temperature, precipitation, wind patterns, changes in seasons, and extreme weather events or anomalies (such as droughts, hurricanes, wildfires, landslides, floods, and tornados, among others).

The articles’ second most addressed aspect of perception was the observation of human phenomena. Thus, in 85% of the articles (n = 309), changes in subsistence practices were observed (such as modifications in the timing of activities or the abandonment of certain practices; changes in human health (e.g., increased disease incidence, basic sanitation needs); alterations in food availability; human migration patterns; or the acquisition/loss of traditional knowledge.

Lastly, perception of biological phenomena was reported in 70% of the articles (n = 254). These articles presented whether changes were observed or not in plants (e.g., alternations in vegetation cover, the appearance of invasive species, and flowering patterns of specific plants), animals (e.g., the emergence or disappearance of particular species, disappearance of insect populations, the emergence of pests, and the presence of invasive species), and other living beings.

### Limitations of the map

One of the limitations of this study is the English-only document review, which is justified by three reasons. First, the article's primary objective was to identify and describe the various perception concepts in use rather than attempting to analyze each individual article that adopted the term. Second, specific perception terms (e.g., awareness, attitudes) lack direct translation across languages and, consequently, including other idioms would introduce inconsistencies in the coding process. Third, our pre-tests did not reveal any significant geographical distribution in using these concepts. Our choice provides access to worldwide research while reducing selection bias that could result from focusing on a few languages. Furthermore, as the primary goal of this article is to systematize and analyze the alternative uses of the term “perception” in the context of climate change, language differences would likely pose insurmountable challenges to our review, particularly when dealing with implicit definitions.

A second limitation is the restriction of the search period to 2018–2022. Expanding the sample to include years before 2018 could be valuable for two reasons. First, it would allow the inclusion of more articles concerning small-scale populations, as indicated in our published protocol [85]. Second, it would enable comparisons between recent and older literature, facilitating the evaluation of trends.

Third, although the review team devoted effort to the elaboration of the search string (see our protocol [85]), the string may not have captured all existing literature on the subject for reasons we are not aware of.

Fourth, owing to the considerable volume of articles on one hand and our limited time and resources on the other, we did not review the grey literature in this study. While this might be considered a limitation to be addressed by other researchers in future studies, we have no reason to believe that the patterns of concept usage differ between these two types of literature.

Lastly, because in most articles’ definitions were implicit, we may have missed a few definitions or constructs even if attempting to encompass all the spectrum of concepts.

## Conclusions

This systematic mapping provided a detailed description of how the concept of climate change perception has been adopted, both explicitly or implicitly, in the scientific literature. After analyzing 361 articles (out of 5,358 screened), we observed that the definitions of climate change perception for small-scale populations varied when explicit. Explicit definitions fell into seven main categories: risk perception, perception according to psychological constructs and sensory stimuli, awareness, prior experience, observation of climate variables, beliefs and interpretations of the environment, and uncertainties and threats. However, explicit concepts were relatively scarce, comprising only one-sixth of the total articles. As a result, the literature primarily consists of implicit uses of the concept, which we have analyzed by identifying constructs within the articles.

As for explicit and implicit definitions, we initially expected that most concepts would align with Cognitive Psychology theories. However, we found that the concepts were interdisciplinary in nature. For instance, perception was defined not only based on psychological and sensory constructs but also through direct experiences with the phenomenon, indirect experiences, sets of beliefs, worldviews, and traditional knowledge. Furthermore, authors often began their texts by defining perception as a sensory stimulus, for example. However, when presenting results, authors frequently referred to perception in different ways, such as an individual's concern about climate change or a set of beliefs.

The explicit definitions were diverse, encompassing various thematic areas and perception constructs. Many articles addressed multiple subjects or perception constructs, although there were challenges in identifying the perception construct used, as they were often implicit. Consequently, other constructs may have been unintentionally overlooked by the reviewers.

This conceptual confusion is somehow expected, as climate change is not solely defined by perception and awareness. Frequently, other factors are essential for understanding it, including risk perception, individuals’ beliefs about climate change, and their direct or indirect exposure to its effects.

When it comes to a comprehensive theoretical understanding of smallholders’ perceptions of climate change, the findings suggest that the literature requires improvement. Even in cases where articles presented a theory throughout their content, the analysis was not consistently guided by that theory when assessing study results. In many other articles, theories were only mentioned sporadically. For example, theories might only appear in the methods section or the final discussion, without having guided the data collection process. As expected, when theoretical or analytical frameworks were adopted, particularly the Capital Approach Framework or Cognitive Psychology theories, it facilitated easier comparisons of results across studies.

We can speculate about why the literature has not yet reached a high level of conceptual and theoretical advancement. One possible reason is the field's interdisciplinary nature, with researchers who address the concept of perception often coming from various disciplines and, sometimes, with academic backgrounds outside of Psychology and other Social Sciences. Consequently, they may not always adhere to the rigorous protocols in these scientific fields, where clear concept presentation and consistent definitions throughout the papers are crucial. These shortcomings in current research on smallholders’ perceptions could potentially impede progress in accumulating knowledge and, consequently, hinder the development of effective public policies to mitigate the negative impacts of climate change on vulnerable populations.

### Implication for policy/management

We highlight three key implications for policy development.

Firstly, it is essential to foster a comprehensive understanding of the concept of climate change perception. Its absence can lead to erroneous conclusions, limited adaptation strategies, and a failure to comprehend the full spectrum of climate change impacts. For example, suppose most studies focus on perception solely as a measure of direct observation of current natural disasters. In that case, adaptation measures may overlook more subtle changes with potentially more severe long-term consequences, such as shifts in agricultural productivity. Given that climate change can introduce new impacts over time, a lack of perception hampers the ability to adapt to evolving climate conditions.

Secondly, gaining a clearer understanding of conceptual differences in perceptions is crucial because researchers' conceptualizations of perception influence data collection methods, potentially affecting research outcomes and, in turn, policy formulation. Once again, the misuse or misunderstanding of these concepts can lead to ineffective policies.

Lastly, the absence of clear definitions for the concept of perception and its associated constructs may impede the effective engagement of small-scale communities in participatory research and the implementation of adaptation strategies. Small-scale communities may have differing interpretations of the concept and its constructs compared to researchers. For instance, alternative ways of perceiving gradual changes or perceptions that are more subjective than objective may be overlooked. In all cases, without rigorously adopting definitions suitable for smallholders, discussions about impacts and adaptation may inadvertently exclude the most vulnerable and at-risk small-scale populations.

### Implication for research

There are four main implications for research.

First, the field of climate change research is inherently interdisciplinary. Therefore, maintaining a continuous dialogue among various disciplines is essential for developing one or more interdisciplinary definitions of the perception concept. This collaboration allows researchers from diverse fields to pursue their studies based on their unique perspectives on perception while also promoting integration across disciplines.

Second, defining the concepts of perception and its associated constructs is crucial, as this is essential for enabling comparisons of results across studies. Attempting to compare results from articles on climate change perceptions becomes impractical or misleading when (i) the concept of perception is left undefined, (ii) different perception definitions are employed, or (iii) comparisons are made between articles that share constructs but use different definitions for the term.

Third, it is probable that many facets of perception are being overlooked, even though implicit constructs are often utilized in defining perception. For instance, authors often allude to direct experience in their articles’ results, even when employing different terminology, such as "observing climate change through subsistence activities or engaging in specific practices.” However, the initial definition of perception is often described as sensory stimuli. Thus, it is essential to standardize the concept throughout the text.

Lastly, more research in Central and Latin America focusing on small-scale populations is needed, as our analysis has revealed a lack of investigations in these two regions. These areas are home to various small-scale populations, which are particularly vulnerable to the impacts of climate change, including indigenous and non-indigenous communities with varying levels of dependence on natural resources, residing in diverse ecosystems, both urban and remote rural. Additionally, our description of the thematic areas explored serves as a valuable evidence database for researchers seeking to identify areas requiring further scientific investigations in small-scale populations.

## Supplementary Information

**Additional file 1.** ROSES checklist of systematic map protocol.

**Additional file 2. **Codebook—Description of the data that will be extracted in the systematic map.

**Additional file 3.** Record of all articles.

**Additional file 4.** Complete list of explicit definitions of perception.

**Additional file 5.** Complete reference list of the articles selected for full reading.

**Additional file 6.** Complete list of articles excluded in the title and abstract screening phase and the reasons for exclusion.

**Additional file 7.** Data extracted from 361 articles after reading in full.

**Additional file 8.** Percentage of thematic areas covered in the 361 articles.

**Additional file 9.** Information on the theories presented in the articles.

**Additional file 10.** Information about constructs (n = 361).

**Additional file 11.** Constructs by geographical distribution.

**Additional file 12.** Complete list of the description of how perception shows up in results.

## Data Availability

Not applicable.

## Footnotes

1 Russia is located in two continents (Europa and Asia). Here it is considered part of the European continent, as the Codebook defines it as “*transcontinental—generally considered European*.”.
