# Supplementary material for: Existing evidence of conceptual differences in research on climate change perceptions among smallholders? A systematic map
Source: Environ Evid. 2023 Dec 7;12:28. doi: 10.1186/s13750-023-00321-2 (PMC11378835; doi:10.1186/s13750-023-00321-2)
Supplement: Supplementary file 8 — Additional file 8. Percentage of thematic areas covered in the 361 articles. [file 13750_2023_321_MOESM8_ESM.docx]

**Additional file 8** Percentage of thematic areas covered in the 361 articles

| **Thematic area covered in the article** | **Proportion and frequency**  **(% (n))** | **Examples** | **Reference examples** |
| --- | --- | --- | --- |
| Changes observed in the environment, e.g. description of changes and resulting environmental impacts. | 97% (349) | Changes observed in the environment. | (1–6) |
|  |  | Environmental impacts of climate change. | (2,5,7–11) |
| Changes observed in livelihood through: (i) description of changes and their impacts on people's livelihoods (e.g. agricultural productivity, monetary income, and others.) or (ii) vital aspects for human survival. | 94% (340) | Changes and impacts on livelihood through the Sustainable Livelihoods Approach (SLA): analyzing capital (human, social, physical, natural, and financial). | (12–19) |
|  |  | Changes and impacts on livelihood without analyzing forms of capital (human, social, physical, natural, and financial). | (4,14,20–26) |
|  |  | Changes and impacts on livelihoods through the perception of climate risks. | (27) |
| Adaptation, including measures, initiatives, and coping strategies (adopted or planned) to reduce or prevent current and/or expected adverse effects caused by climate change. Independent of the actor who implements them. | 74% (267) | Determinants of adaptation (e.g. education, experience, climate change perception, information, and others). | (28–36) |
|  |  | Adoption of different adaptation measures | (7,37–42) |
|  |  | Ecosystem-based Adaptation approach | (21,43) |
|  |  | Climate-smart agriculture | (10,44–49) |
|  |  | Coping strategies | (2,50–55) |
| Scientific knowledge: presentation of meteorological or physical data or any other type of information gathered by scientists and researchers. | 57% (206) | Presentation of meteorological data (e.g. trends, historical temperature data, precipitation). | (38,56–62) |
| Comparison between the observations of investigated individuals and scientific data, such as meteorological data on climate variability and/or climate anomalies. | 55% (197) | Comparison between the perception of individuals and meteorological data. | (57,59,63–69) |
| Awareness, i.e. the state or ability to perceive, feel, or be aware of events, objects, or sensory patterns relative to climate change. | 44% (157) | Awareness of climate change | (66,70–74) |
|  |  | Factors affecting awareness (e.g. education, experience, access to information, monetary income, and others). | (71,73) |
| Traditional knowledge | 31% (112) | Traditional knowledge: identification of physical indicators (e.g. clouds, star position, atmospheric aspects) and/or biological (e.g. animal behavior, the flowering of plants, presence of insects), or others. | (75–78) |
|  |  | Traditional knowledge without the identification of indicators. | (21,79–82) |
| Resilience, i.e. the ability of social, economic, and environmental systems to respond to or cope with dangerous events, trends, or any disturbance. To preserve functions, identities, and essential structures, while maintaining capacities for adaptation, learning, and transformation. | 25% (89) | Resilience of capital (human, social, physical, natural, and financial). | (16,83) |
|  |  | The resilience of socioecological systems. | (84) |
|  |  | Livelihood resilience. | (27,85,86) |
|  |  | Resilience to the impacts of climate change (e.g. drought). | (87–89) |
| Determinant factors of perception, such as age, sex, education, monetary income, and access to information. | 21% (76) | Determinants of perception (e.g. age, sex, access to information; access to credit, household size, and others). | (62,90–98) |
|  |  | Determinants of risk perception (e.g. gender, experience). | (55,99–101) |
| Risk perception, i.e. subjective judgment of people, communities, or households about the characteristics and severity of climate change impacts. | 13% (46) | Climate risk perception. | (29,37,55,99,102–108) |
|  |  | Climate-Related Risk Perception Index; Climate Change Risk Perception Index. | (109–112) |
|  |  | Risk perception based on Prospect Theory. | (113) |
|  |  | Gender and risk perception. | (114) |
| Mitigation refering to technological changes and/or other types of replacements that reduce greenhouse gas emissions or that act to reduce these gases and aerosols. | 12% (44) | Adaptation strategies to mitigate adverse effects of climate change. | (64,106) |
|  |  | Mitigation strategy. | (3,45,110,115,116) |
| Other topics | 11% (41) | Non-timber forest products. | (117,118) |
|  |  | Tourism | (119,120) |
|  |  | Prospect theory, heuristics, psychological barriers, memory, mental model, cogeneration of knowledge. | (27,113,121–124) |
|  |  | Forest area; land use; agrobiodiversity; agroforestry, conservation agriculture, environmental values; | (107,125–129) |
|  |  | Vegetables or açaí cultivation. | (93,130–132) |
|  |  | El Niño; hurricane, pests, wildfire, animal disease, cryospheric hazards, climate shock. | (85,103,133–137) |
|  |  | Poverty | (17,138) |
|  |  | Climate governance, risk management, trust in the institution. | (105,139,140) |
|  |  | Keystone species; phenological events, ecosystem services of mangroves. | (79,141,142) |
|  |  | Socioecological system. | (18,143) |
|  |  | Religious beliefs. | (144) |
|  |  | Water quality; watershed development programs. | (33,145) |
| Vulnerability, i.e. predisposition of individuals, communities, or households to be adversely affected by climate change. | 7% (26) | Vulnerability assessment (Climate Vulnerability Index) | (50,109,110,146,147) |
|  |  | Vulnerability context in climate change | (20,37,76,148,149) |
|  |  | Vulnerability level | (150) |
|  |  | Vulnerability matrix | (151,152) |
|  |  | Livelihood vulnerability index | (153,154) |
|  |  | Gender-based vulnerability | (155) |
| Health, food security, and nutrition. | 3% (10) | Influence of climate change on health | (156–158) |
|  |  | Mental health | (159) |
|  |  | Food (in)security | (55,86,149,160,161) |
|  |  | Children's nutrition | (156) |
|  |  | Food consumption patterns | (162) |
| Gender issues related to climate change perception. | 2% (7) | Gender, practice of subsistence activity, and adaptation strategy. | (163) |
|  |  | Gender and influence on risk perception, awareness, and adaptation strategy. | (114) |
|  |  | Gender and climate change perception | (16,155,164,165) |
|  |  | Women and climate change perception, awareness, and adaptation strategies. | (166,167) |

**References**

1. Kuniyal JC, Kanwar N, Bhoj AS, Rautela KS, Joshi P, Kumar K, et al. Climate Change Impacts on Glacier-fed and Non-glacier-Fed Ecosystems of the Indian Himalayan Region: People’s Perception and Adaptive Strategies. Curr Sci. 2021;120(5):888.

2. Mekuyie M, Mulu D. Perception of Impacts of Climate Variability on Pastoralists and Their Adaptation/Coping Strategies in Fentale District of Oromia Region, Ethiopia. Environmental Systems Research. 2021;10(1):4.

3. Samuel KJ, Adeniyi SA, Adetunji K. Knowledge and Perception of Climate Change among Peasant Farmers in a Forest-Savannah Transition Zoneof Osun State, Nigeria. African Journal of Sustainable Development. 2018;7(1).

4. Ludago SC, Amanuel TW. Socio-economic Impacts of Climate Change on Smallholder Livelihoods in Shashogo District, Hadiya Zone, South Ethiopia. Journal of Environment and Earth Science. 2018;8(2):25–38.

5. Khanal P, Wagle BH, Upadhaya S, Ghimire P, Acharya S. Perceived Climate Change Impacts and Adaptation Strategy of Indigenous Community (Chepangs) in Rural Mid-hills of Nepal. Foresty Journal of Institute of Forestry. 2019;(16):48–61.

6. Ayinde AFO, Johnston PA, Olujimi OO, Dasgupta P, Akerele D. Perception of Cassava-Based Farmers to Climate Variability in the Rain Forest and Derived Savannah Biomes of Nigeria. Sarhad Journal of Agriculture. 2022;38(5).

7. Pandey R, Kumar P, Archie KM, Gupta AK, Joshi PK, Valente D, et al. Climate change adaptation in the western-Himalayas: Household level perspectives on impacts and barriers. Ecol Indic. 2018;84:27–37.

8. Popoola OO, Monde N, Yusuf SFG. Perceptions of climate change impacts and adaptation measures used by crop smallholder farmers in Amathole district municipality, Eastern Cape province, South Africa. GeoJournal. 2018;83(6):1205–21.

9. Dawadi B, Shrestha A, Acharya RH, Dhital YP, Devkota R. Impact of climate change on agricultural production: A case of Rasuwa District, Nepal. Regional Sustainability. 2022;3(2):122–32.

10. Belay A, Oludhe C, Mirzabaev A, Recha JW, Berhane Z, Osano PM, et al. Knowledge of climate change and adaptation by smallholder farmers: evidence from southern Ethiopia. Heliyon. 2022;8(12):e12089.

11. Anteneh M. Climate Variability Patterns and Farmers’ Perceptions of Its Impact on Food Production: A Case Study of the  Gelda Watershed in the Lake Tana Basin in Northwest  Ethiopia. Air, Soil and Water Research. 2022;15:1–11.

12. Yiridomoh GY, Appiah DO, Owusu V, Bonye SZ. Women smallholder farmers off-farm adaptation strategies to climate variability in rural Savannah, Ghana. GeoJournal. 2021;86(5):2367–85.

13. Ndlovu E, Prinsloo B, le Roux T. Impact of climate change and variability on traditional farming systems: Farmers’ perceptions from south-west, semi-arid Zimbabwe. Jàmbá: Journal of Disaster Risk Studies. 2020;12(1).

14. Monwar MdM, Mustafa MdG, Khan NA, Hossain MS, Hossain MM, Majumder MK, et al. Indigenous Adaptation Practices for the Development of Climate Resilient Ecosystems in the Hail Haor, Bangladesh. Global Social Welfare. 2018;5(2):125–36.

15. Žurovec O, Vedeld PO. Rural Livelihoods and Climate Change Adaptation in Laggard Transitional Economies: A Case from Bosnia and Herzegovina. Sustainability. 2019;11(21):6079.

16. Quandt A. Variability in perceptions of household livelihood resilience and drought at the intersection of gender and ethnicity. Clim Change. 2019;152(1):1–15.

17. Torres B, Cayambe J, Paz S, Ayerve K, Heredia-R M, Torres E, et al. Livelihood Capitals, Income Inequality, and the Perception of Climate Change: A Case Study of Small-Scale Cattle Farmers in the Ecuadorian Andes. Sustainability. 2022;14(9):5028.

18. Bauer TN, de Jong W, Ingram V. Perception matters: an Indigenous perspective on climate change and its effects on forest-based livelihoods in the Amazon. Ecology and Society. 2022;27(1):art17.

19. Guáqueta-Solórzano VE, Postigo JC. Indigenous perceptions and adaptive responses to the impacts of climate variability in the Sierra Nevada de Santa Marta, Colombia. Frontiers in Climate. 2022;4.

20. Kieslinger J, Pohle P, Buitrón V, Peters T. Encounters Between Experiences and Measurements: The Role of Local Knowledge in Climate Change Research. Mt Res Dev. 2019;39(2).

21. Kupika OL, Gandiwa E, Nhamo G, Kativu S. Local Ecological Knowledge on Climate Change and Ecosystem-Based Adaptation Strategies Promote Resilience in the Middle Zambezi Biosphere Reserve, Zimbabwe. Scientifica (Cairo). 2019;2019:1–15.

22. Saalu FN, Oriaso S, Gyampoh B. Effects of a changing climate on livelihoods of forest dependent communities. Int J Clim Chang Strateg Manag. 2020;12(1):1–21.

23. Singh RK, Singh A, Kumar S, Sheoran P, Sharma DK, Stringer LC, et al. Perceived Climate Variability and Compounding Stressors: Implications for Risks to Livelihoods of Smallholder Indian Farmers. Environ Manage. 2020;66(5):826–44.

24. Nef DP, Neneth D, Dini P, Abad CR, Kruetli P. How local communities attribute livelihood vulnerabilities to climate change and other causes: a case study in North Vanuatu. Clim Change. 2021;168(3–4):17.

25. Mashara JN. Climate Change Perception Among the Pastoralist Women in Narok County. Academic Research International. 2018;9(1):147–53.

26. Tugjamba N, Walkerden G, Miller F. Climate change impacts on nomadic herders’ livelihoods and pastureland ecosystems: a case study from Northeast Mongolia. Reg Environ Change. 2021;21(4):105.

27. Sharma R, Jagtap S, Rao P. Understanding Maharashtra Coastal Community’s Perceptions and Livelihood Resilience to Climate Change Using the Community Participatory Approach. The International Journal of Climate Change: Impacts and Responses. 2022;14(2):1–19.

28. Ceci P, Monforte L, Perelli C, Cicatiello C, Branca G, Franco S, et al. Smallholder farmers’ perception of climate change and drivers of adaptation in agriculture: A case study in Guinea. Rev Dev Econ. 2021;25(4):1991–2012.

29. Baloch ZA, Tan Q, Fahad S. Analyzing farm households’ perception and choice of adaptation strategies towards climate change impacts: a case study of vulnerable households in an emerging Asian region. Environmental Science and Pollution Research. 2022;29(38):57306–16.

30. Musafiri CM, Kiboi M, Macharia J, Ng’etich OK, Kosgei DK, Mulianga B, et al. Smallholders’ adaptation to climate change in Western Kenya: Considering socioeconomic, institutional and biophysical determinants. Environmental Challenges. 2022;7:100489.

31. Vo HH, Mizunoya T, Nguyen CD. Determinants of farmers’ adaptation decisions to climate change in the central coastal region of Vietnam. Asia-Pacific Journal of Regional Science. 2021;5(2):327–49.

32. Mesfin AH, Bekele A. Farmers Perception on Climate Change and Determinants of Adaptation Strategies in Benishangul-Gumuz Regional State of Ethiopia. Int J Food System Dynamics. 2018;9(5):453–69.

33. Funk C, Raghavan Sathyan A, Winker P, Breuer L. Changing climate - Changing livelihood: Smallholder’s perceptions and adaption strategies. J Environ Manage. 2020;259:109702.

34. Kangai R, Wemali Chitechi E, Koske J, Boaz W, Innocent N. Determinants of climate change adaptation and perceptions among small-scale farmers of Embu County, Eastern Kenya. Afr J Environ Sci Tech. 2021;15(4):167–78.

35. Guodaar L, Appiah DO. Evolving farm-level adaptation to climate variability and change risks in the forest-savanna transitional zone of Ghana. Environmental Challenges. 2022;9:100654.

36. Zeleke T, Beyene F, Deressa T, Yousuf J, Kebede T. Smallholder farmers’ perception of climate change and choice of adaptation strategies in East Hararghe Zone, Eastern Ethiopia. Int J Clim Chang Strateg Manag. 2022;22.

37. Twecan D, Wang W, Xu J, Mohmmed A. Climate change vulnerability, adaptation measures, and risk perceptions at households level in Acholi sub-region, Northern Uganda. Land use policy. 2022;115:106011.

38. Cuni-Sanchez A, Omeny P, Pfeifer M, Olaka L, Mamo MB, Marchant R, et al. Climate change and pastoralists: perceptions and adaptation in montane Kenya. Clim Dev. 2019;11(6):513–24.

39. Rai S, Dahal B, Anup KC. Climate change perceptions and adaptations by indigenous Chepang community of Dhading, Nepal. GeoJournal. 2022;16.

40. Timité N, Kouakou ATM, Bamba I, Barima YSS, Bogaert J. Climate Variability in the Sudanian Zone of Côte d’Ivoire: Weather Observations, Perceptions, and Adaptation Strategies of Farmers. Sustainability. 2022;14(16):10410.

41. Kouassi JL, Wandan N, Mbow C. Observed climate trends, perceived impacts and community adaptation practices in Côte d’Ivoire. Environmental & Socio-economic Studies. 2022;10(3):43–58.

42. Landaverde R, Rodriguez MT, Niewoehner-Green J, Kitchel T, Chuquillanqui J. Climate Change Perceptions and Adaptation Strategies: A Mixed Methods Study with Subsistence Farmers in Rural Peru. Sustainability. 2022;14(23):16015.

43. Inman EN, Hobbs RJ, Tsvuura Z. No safety net in the face of climate change: The case of pastoralists in Kunene Region, Namibia. PLoS One. 2020;15(9):e0238982.

44. Ali E. Farm Households’ Adoption of Climate-smart Practices in Subsistence Agriculture: Evidence from Northern Togo. Environ Manage. 2021;67(5):949–62.

45. Mairura FS, Musafiri CM, Kiboi MN, Macharia JM, Ng’etich OK, Shisanya CA, et al. Determinants of farmers’ perceptions of climate variability, mitigation, and adaptation strategies in the central highlands of Kenya. Weather Clim Extrem. 2021;34:1–14.

46. Autio A, Johansson T, Motaroki L, Minoia P, Pellikka P. Constraints for adopting climate-smart agricultural practices among smallholder farmers in Southeast Kenya. Agric Syst. 2021;194:103284.

47. Nyang’au JO, Mohamed JH, Mango N, Makate C, Wangeci AN. Smallholder farmers’ perception of climate change and adoption of climate smart agriculture practices in Masaba South Sub-county, Kisii, Kenya. Heliyon. 2021;7(4):e06789.

48. Adhikari S, Rawal S, Thapa S. Assessment of Status of Climate Change and Determinants of People’s Awareness to Climate-Smart Agriculture: A Case of Sarlahi District, Nepal. Advances in Agriculture. 2022;2022:1–9.

49. Moutouama FT, Tepa-Yotto GT, Agboton C, Gbaguidi B, Sekabira H, Tamò M. Farmers’ Perception of Climate Change and Climate-Smart Agriculture in Northern Benin, West Africa. Agronomy. 2022;12(6):1348.

50. Limuwa M, Sitaula B, Njaya F, Storebakken T. Evaluation of Small-Scale Fishers’ Perceptions on Climate Change and Their Coping Strategies: Insights from Lake Malawi. Climate. 2018;6(2):34.

51. Tunde AM, Ajadi BS. Indigenous understanding of climate change, impacts and coping strategies in a rural setting of Kwara State, Nigeria. Geography, Environment, Sustainability. 2019;11(4):85–99.

52. Ahmed MNQ, Atiqul Haq SM. Indigenous people’s perceptions about climate change, forest resource management, and coping strategies: a comparative study in Bangladesh. Environ Dev Sustain. 2019;21(2):679–708.

53. Staub C, Gilot A, Pierre M, Murray G, Koenig R. Coping with climatic shocks: local perspectives from Haiti’s rural mountain regions. Popul Environ. 2020;42(2):146–58.

54. Gebeyehu AK, Snelder D, Sonneveld B, Abbink J. How do agro-pastoralists cope with climate change? The case of the Nyangatom in the Lower Omo Valley of Ethiopia. J Arid Environ. 2021;189:104485.

55. Kanwal V, Sirohi S, Chand P. Farmers’ perception on climate extremes and their coping mechanism: evidences from disaster prone regions of India. Indian Journal of Traditional Knowledge. 2021;20(2).

56. Funatsu BM, Dubreuil V, Racapé A, Debortoli NS, Nasuti S, le Tourneau FM. Perceptions of climate and climate change by Amazonian communities. Global Environmental Change. 2019;57:101923.

57. Behailu G, Ayal DY, Zeleke TT, Ture K, Bantider A. Comparative Analysis of Meteorological Records of Climate Variability and Farmers’ Perceptions in Sekota Woreda, Ethiopia. Clim Serv. 2021;23:100239.

58. Hamal R, Thakuri BM, Poudel KR, Gurung A, Yun SJ. Farmers’ perceptions of climate change in Lower Mustang, Nepal. Environ Monit Assess. 2022;194(9):606.

59. Mkonda MY, He X, Festin ES. Comparing Smallholder Farmers’ Perception of Climate Change with Meteorological Data: Experience from Seven Agroecological Zones of Tanzania. Weather, Climate, and Society. 2018;10(3):435–52.

60. Mubiru DN, Radeny M, Kyazze FB, Zziwa A, Lwasa J, Kinyangi J, et al. Climate trends, risks and coping strategies in smallholder farming systems in Uganda. Clim Risk Manag. 2018;22:4–21.

61. Joshi A, Farquhar S, Assareh N, Dahlet L, Landahl E. Climate change in Lamjung District, Nepal: meteorological evidence, community perceptions, and responses. Environ Res Commun. 2019;1(3):031004.

62. Datta P, Behera B. Do farmers perceive climate change clearly? An analysis of meteorological data and farmers’ perceptions in the sub-Himalayan West Bengal, India. Journal of Water and Climate Change. 2022;13(5):2188–204.

63. Darabant A, Habermann B, Sisay K, Thurnher C, Worku Y, Damtew S, et al. Farmers’ perceptions and matching climate records jointly explain adaptation responses in four communities around Lake Tana, Ethiopia. Clim Change. 2020;163(1):481–97.

64. Roy D, Datta A, Kuwornu JKM, Zulfiqar F. Comparing farmers’ perceptions of climate change with meteorological trends and examining farm adaptation measures in hazard-prone districts of northwest Bangladesh. Environ Dev Sustain. 2021;23(6):8699–721.

65. Kamruzzaman Md, Rahman ATMS, Basak A, Alam J, Das J. Assessment and adaptation strategies of climate change through the prism of farmers’ perception: A case study. International Journal of Environmental Science and Technology. 2022;20.

66. Ocak Yetisgin S, Önder H, Şen U, Piwczyński D, Kolenda M, Sitkowska B, et al. Farmers’ Risk Perception on Climate Change: Transhumance vs. Semi-Intensive Sheep Production Systems in Türkiye. Animals. 2022;12(15):1992.

67. Muringai RT, Mafongoya P, Lottering RT. Climate Change Perceptions, Impacts and Adaptation Strategies: Insights of Fishers in Zambezi River Basin, Zimbabwe. Sustainability. 2022;14(6):3456.

68. Abazinab H, Duguma B, Muleta E. Livestock farmers’ perception of climate change and adaptation strategies in the Gera district, Jimma zone, Oromia Regional state, southwest Ethiopia. Heliyon. 2022;8(12):e12200.

69. Paramesh V, Kumar P, Shamim M, Ravisankar N, Arunachalam V, Nath AJ, et al. Integrated Farming Systems as an Adaptation Strategy to Climate Change: Case Studies from Diverse Agro-Climatic Zones of India. Sustainability. 2022;14(18):11629.

70. Jibrillah AM, Jaafara M, Choy LK. Climate Change Awareness and Adaptations Among the Farming and Animal Rearing Communities of the Central Sokoto Close-Settle Zone, North-Western Nigeria. Jurnal Kejuruteraan SI. 2018;1(6):71–9.

71. Ado AM, Leshan J, Savadogo P, Bo L, Shah AA. Farmers’ awareness and perception of climate change impacts: case study of Aguie district in Niger. Environ Dev Sustain. 2019;21(6):2963–77.

72. Hundera H, Mpandeli S, Bantider A. Smallholder farmers’ awareness and perceptions of climate change in Adama district, central rift valley of Ethiopia. Weather Clim Extrem. 2019;26:100230.

73. Pondorfer A. The perception of climate change: Comparative evidence from the small-island societies of Bougainville and Palawan. Environ Dev. 2019;30:21–34.

74. Ng’ombe JN, Tembo MC, Masasi B. “Are They Aware, and Why?” Bayesian Analysis of Predictors of Smallholder Farmers’ Awareness of Climate Change and Its Risks to Agriculture. Agronomy. 2020;10(3):376.

75. Tume SJP, Kimengsi JN, Fogwe ZN. Indigenous Knowledge and Farmer Perceptions of Climate and Ecological Changes in the Bamenda Highlands of Cameroon: Insights from the Bui Plateau. Climate. 2019;7(12):138.

76. Maliki MA, Pauline NM. Living and Responding to Climatic Stresses: Perspectives from Smallholder Farmers in Hanang’ District, Tanzania. Environ Manage. 2022;14.

77. Mekonnen Z, Kassa H, Woldeamanuel T, Asfaw Z. Analysis of observed and perceived climate change and variability in Arsi Negele District, Ethiopia. Environ Dev Sustain. 2018;20(3):1191–212.

78. Inaotombi S, Mahanta PC. Pathways of socio-ecological resilience to climate change for fisheries through indigenous knowledge. Human and Ecological Risk Assessment: An International Journal. 2019;25(8):2032–44.

79. Wyllie de Echeverria VR, Thornton TF. Using traditional ecological knowledge to understand and adapt to climate and biodiversity change on the Pacific coast of North America. Ambio. 2019;48(12):1447–69.

80. Tumenjargal S, Fassnacht SR, Venable NBH, Kingston AP, Fernández-Giménez ME, Batbuyan B, et al. Variability and change of climate extremes from indigenous herder knowledge and at meteorological stations across central Mongolia. Front Earth Sci. 2020;14(2):286–97.

81. Miara MD, Negadi M, Tabak S, Bendif H, Dahmani W, Ait Hammou M, et al. Climate Change Impacts Can Be Differentially Perceived Across Time Scales: A Study Among the Tuareg of the Algerian Sahara. Geohealth. 2022;6(11).

82. Estevo M de O, Junqueira AB, Reyes-García V, Campos-Silva JV. Understanding Multidirectional Climate Change Impacts on Local Livelihoods through the Lens of Local Ecological Knowledge: A Study in Western Amazonia. Soc Nat Resour. 2022;1–18.

83. Monwar MdM, Mustafa MdG, Khan NA, Hossain MS, Hossain MM, Majumder MK, et al. Indigenous Adaptation Practices for the Development of Climate Resilient Ecosystems in the Hail Haor, Bangladesh. Global Social Welfare. 2018;5(2):125–36.

84. Córdoba Vargas CA, Hortúa Romero S, León-Sicard T. Resilience to climate variability: the role of perceptions and traditional knowledge in the Colombian Andes. Agroecology and Sustainable Food Systems. 2020;44(4):419–45.

85. Metcalfe SE, Schmook B, Boyd DS, de la Barreda-Bautista B, Endfield GE, Mardero S, et al. Community perception, adaptation and resilience to extreme weather in the Yucatan Peninsula, Mexico. Reg Environ Change. 2020;20(1):25.

86. al Dirani A, Abebe GK, Bahn RA, Martiniello G, Bashour I. Exploring climate change adaptation practices and household food security in the Middle Eastern context: a case of small family farms in Central Bekaa, Lebanon. Food Secur. 2021;13(4):1029–47.

87. Bahta YT. Perception of agricultural drought resilience in South Africa: A case of smallholder livestock farmers. Jàmbá Journal of Disaster Risk Studies. 2021;13(1).

88. Baig SM, Khan AA, Ali A, Khan MZ, Ahmed S, Shah GM, et al. Enhancing socioeconomic resilience and climate adaptation through value chain development of mountain products in Hindu Kush Himalayas. Environ Dev Sustain. 2021;23(6):8451–73.

89. Habte M, Eshetu M, Maryo M, Andualem D, Legesse A. Effects of climate variability on livestock productivity and pastoralists perception: The case of drought resilience in Southeastern Ethiopia. Vet Anim Sci. 2022;16:100240.

90. Guodaar L, Bardsley DK, Suh J. Integrating local perceptions with scientific evidence to understand climate change variability in northern Ghana: A mixed-methods approach. Applied Geography. 2021;130:102440.

91. Escarcha JF, Lassa JA, Palacpac EP, Zander KK. Understanding climate change impacts on water buffalo production through farmers’ perceptions. Clim Risk Manag. 2018;20:50–63.

92. Sraku-Lartey M, Buor D, Adjei POW, Foli EG. Perceptions and knowledge on climate change in local communities in the Offinso Municipality, Ghana. Information Development. 2020;36(1):16–35.

93. Fadairo O, Williams PA, Nalwanga FS. Perceived livelihood impacts and adaptation of vegetable farmers to climate variability and change in selected sites from Ghana, Uganda and Nigeria. Environ Dev Sustain. 2020;22(7):6831–49.

94. Hoang HG. Vietnamese smallholders’ perspectives on causes, indicators and determinants of climate change: implication for adaptation strategies. Clim Change. 2020;162(3):1127–42.

95. Negi VS, Thakur S, Dhyani R, Bhatt ID, Rawal RS. Climate Change Observations of Indigenous Communities in the Indian Himalaya. Weather, Climate, and Society. 2021;13(2):245–57.

96. Hasan MK, Kumar L. Changes in coastal farming systems in a changing climate in Bangladesh. Reg Environ Change. 2022 Dec 6;22(4):113.

97. Xie S, Ding W, Ye W, Deng Z. Agro-pastoralists’ perception of climate change and adaptation in the Qilian Mountains of northwest China. Sci Rep. 2022;12(1):12689.

98. Awoke W, Agitew G. Rural households’ perception of climate change in the central and north Gondar zones, northwest Ethiopia. African Journal of Food, Agriculture, Nutrition and Development. 2022;22(113):21362–82.

99. Smith RA. Risk perception and adaptive responses to climate change and climatic variability in northeastern St. Vincent. J Environ Stud Sci. 2018;8(1):73–85.

100. Magalhães HF, Feitosa IS, de Lima Araújo E, Albuquerque UP. Perceptions of Risks Related to Climate Change in Agroecosystems in a Semi-arid Region of Brazil. Hum Ecol. 2021;49(4):403–13.

101. Budhathoki NK, Paton D, A. Lassa J, Zander KK. Assessing farmers’ preparedness to cope with the impacts of multiple climate change-related hazards in the Terai lowlands of Nepal. International Journal of Disaster Risk Reduction. 2020;49:101656.

102. Guodaar L, Bardsley DK, Suh J. Indigenous adaptation to climate change risks in northern Ghana. Clim Change. 2021;166(1–2):24.

103. Pandey VP, Sharma A, Dhaubanjar S, Bharati L, Joshi IR. Climate Shocks and Responses in Karnali-Mahakali Basins, Western Nepal. Climate. 2019;7(7):92.

104. Raihan ML, Basu M, Onitsuka K, Hoshino S. Determinants of Farmers’ Risk Perceptions of Hailstorms in Northern Bangladesh: Is Adaptive Capacity the Major Concern? Pol J Environ Stud. 2021;31(1):257–70.

105. Tiet T, To-The N, Nguyen-Anh T. Farmers’ behaviors and attitudes toward climate change adaptation: evidence from Vietnamese smallholder farmers. Environ Dev Sustain. 2022;26.

106. Ado AM, Savadogo P, Pervez AKMK, Mudimu GT. Farmers’ perceptions and adaptation strategies to climate risks and their determinants: insights from a farming community of Aguie district in Niger. GeoJournal. 2020;85(4):1075–95.

107. Islam ARMdT, Hasanuzzaman Md, Jaman M, Alam E, Mallick J, Alam GMM, et al. Assessing Farmers’ Typologies of Perception for Adopting Sustainable Adaptation Strategies in Bangladesh. Climate. 2021;9(12):167.

108. Nguyen-Thi-Lan H, Fahad S, Nguyen-Anh T, Tran-Thi-Thu H, Nguyen-Hong C, To-The N. Assessment of farm households’ perception, beliefs and attitude toward climatic risks: A case study of rural Vietnam. PLoS One. 2021;16(12):e0258598.

109. Ahmed Z, Guha GS, Shew AM, Alam GMM. Climate change risk perceptions and agricultural adaptation strategies in vulnerable riverine char islands of Bangladesh. Land use policy. 2021;103:105295.

110. Faisal M, Abbas A, Cai Y, Ali A, Shahzad MA, Akhtar S, et al. Perceptions, Vulnerability and Adaptation Strategies for Mitigating Climate Change Effects among Small Livestock Herders in Punjab, Pakistan. Int J Environ Res Public Health. 2021;18(20):10771.

111. Mamun A al, Roy S, Islam ARMdT, Alam GMM, Alam E, Chandra Pal S, et al. Smallholder Farmers’ Perceived Climate-Related Risk, Impact, and Their Choices of Sustainable Adaptation Strategies. Sustainability. 2021;13(21):11922.

112. Likinaw A, Bewket W, Alemayehu A. Smallholder farmers’ perceptions and adaptation strategies to climate change risks in northwest Ethiopia. Int J Clim Chang Strateg Manag. 2022;20.

113. Villacis AH, Alwang JR, Barrera V. Linking risk preferences and risk perceptions of climate change: A prospect theory approach. Agricultural Economics. 2021;52(5):863–77.

114. Graziano K, Pollnac R, Christie P. Wading past assumptions: Gender dimensions of climate change adaptation in coastal communities of the Philippines. Ocean Coast Manag. 2018;162:24–33.

115. Chidiebere-Mark NM, Ejike RD, Nwaiwu JC, Nwankwo OO, Ibe GO. Assessment of food crop farmers indigenous strategies to climate change mitigation and adaptation in Imo State, Nigeria. Journal of Agriculture and Food Sciences. 2019;16(2):100.

116. Abera N, Tesema D. Perceptions and practices of climate change adaptation and mitigation strategies among farmers in the Konta Special District, Ethiopia. Environmental & Socio-economic Studies. 2019;7(4):1–16.

117. Gurung LJ, Miller KK, Venn S, Bryan BA. Climate change adaptation for managing non-timber forest products in the Nepalese Himalaya. Science of The Total Environment. 2021;796:148853.

118. Gurung LJ, Miller KK, Venn S, Bryan BA. Contributions of non-timber forest products to people in mountain ecosystems and impacts of recent climate change. Ecosystems and People. 2021;17(1):447–63.

119. Saarinen J, Moswete N, Atlhopheng JR, Hambira WL. Changing socio-ecologies of Kalahari: Local perceptions towards environmental change and tourism in Kgalagadi, Botswana. Dev South Afr. 2020;37(5):855–70.

120. Shijin W. Perception of indigenous people of climate change and its impact on the Everest National Nature Preserve. Meteorological Applications. 2021;1–12.

121. Eitzinger A, Binder CR, Meyer MA. Risk perception and decision-making: do farmers consider risks from climate change? Clim Change. 2018;151(3–4):507–24.

122. Waldman KB, Vergopolan N, Attari SZ, Sheffield J, Estes LD, Caylor KK, et al. Cognitive biases about climate variability in smallholder farming systems in Zambia. Weather, Climate, and Society. 2019;11(2):369–83.

123. Dakurah G. How do farmers’ perceptions of climate variability and change match or and mismatch climatic data? Evidence from North-west Ghana. GeoJournal. 2021;86(5):2387–406.

124. Haag I, Kassam KA, Senftl T, Zandler H, Samimi C. Measurements meet human observations: integrating distinctive ways of knowing in the Pamir Mountains of Tajikistan to assess local climate change. Clim Change. 2021;165(1–2):5.

125. Rasmus S, Wallen H, Turunen M, Landauer M, Tahkola J, Jokinen M, et al. Land-use and climate related drivers of change in the reindeer management system in Finland: Geography of perceptions. Applied Geography. 2021;134:102501.

126. Baul TK, Peuly TA, Nandi R, Kar S, Karmakar S. Role of Homestead Forests in Adaptation to Climate Change: A Study on Households’ Perceptions and Relevant Factors in Bandarban Hill District, Bangladesh. Environ Manage. 2022;69(5):906–18.

127. Baffour-Ata F, Antwi-Agyei P, Nkiaka E. Climate Variability, Land Cover Changes and Livelihoods of Communities on the Fringes of Bobiri Forest Reserve, Ghana. Forests. 2021;12(3):278.

128. Njeru EM, Awino RO, Kirui KC, Koech K, Jalloh AA, Muthini M. Agrobiodiversity and perceived climatic change effect on family farming systems in semiarid tropics of Kenya. Open Agric. 2022;7(1):360–72.

129. Paudel D, Tiwari KR, Raut N, Bajracharya RM, Bhattarai S, Sitaula BK, et al. What affects farmers in choosing better agroforestry practice as a strategy of climate change adaptation? An experience from the mid-hills of Nepal. Heliyon. 2022;8(6):e09695.

130. Chepkoech W, Mungai NW, Stöber S, Bett HK, Lotze-Campen H. Farmers’ perspectives. Int J Clim Chang Strateg Manag. 2018;10(4):551–79.

131. Tregidgo D, Campbell AJ, Rivero S, Freitas MAB, Almeida O. Vulnerability of the Açaí Palm to Climate Change. Hum Ecol. 2020;48(4):505–14.

132. Ekemini-Richard M, Ayanwale AB, Adelegan OJ. Factors Influencing Choice of Climate Change Adaptation Methods among Underutilized Indigenous Vegetable farmers. International Journal of Vegetable Science. 2020;28(2):111–20.

133. Sherpa SF, Shrestha M, Eakin H, Boone CG. Cryospheric hazards and risk perceptions in the Sagarmatha (Mt. Everest) National Park and Buffer Zone, Nepal. Natural Hazards. 2019;96(2):607–26.

134. Vinokurova L, Solovyeva V, Filippova V. When Ice Turns to Water: Forest Fires and Indigenous Settlements in the Republic of Sakha (Yakutia). Sustainability. 2022;14(8):4759.

135. Phophi MM, Mafongoya P, Lottering S. Perceptions of Climate Change and Drivers of Insect Pest Outbreaks in Vegetable Crops in Limpopo Province of South Africa. Climate. 2020;8(2):27.

136. Cavole LM, Andrade-Vera S, Marin Jarrin JR, Dias DF, Aburto-Oropeza O, Barrágan-Paladines MJ. Using local ecological knowledge of Fishers to infer the impact of climate variability in Galápagos’ small-scale fisheries. Mar Policy. 2020;121:104195.

137. Ayal DY, Radeny M, Desta S, Gebru G. Climate variability, perceptions of pastoralists and their adaptation strategies. Int J Clim Chang Strateg Manag. 2018;10(4):596–615.

138. Solangi GS, Siyal AA, Siyal Z ul A, Siyal P, Panhwar S, Keerio HA, et al. Social and ecological climate change vulnerability assessment in the Indus delta, Pakistan. Water Pract Technol. 2022;17(8):1666–78.

139. Bekele A, Abebe Y. Choice of Smallholder Farmers’ Adaptation Strategies to Climatic Variability and Their Determinants in Crop Production: The Case of the Central Rift Valley of Ethiopia. Journal of Environment and Earth Science. 2019;9(4):19–28.

140. Mugambiwa SS, Rukema JR. Rethinking indigenous climate governance through climate change and variability discourse by a Zimbabwean rural community. Int J Clim Chang Strateg Manag. 2019;11(5):730–43.

141. Fitchett JM, Ebhuoma E. Phenological cues intrinsic in indigenous knowledge systems for forecasting seasonal climate in the Delta State of Nigeria. Int J Biometeorol. 2018;62(6):1115–9.

142. Nyangoko BP, Berg H, Mangora MM, Shalli MS, Gullström M. Community perceptions of climate change and ecosystem-based adaptation in the mangrove ecosystem of the Rufiji Delta, Tanzania. Clim Dev. 2022;1–13.

143. Ramírez KDI, Ibarra AMA. Less Rain and More Heat”: Smallholders’ Perception and Climate Change Adaptation Strategies in Tropical Environments. Sociedad y Ambiente. 2019;(21):77–104.

144. Scoville-Simonds M. Climate, the Earth, and God – Entangled narratives of cultural and climatic change in the Peruvian Andes. World Dev. 2018;110:345–59.

145. Godebo TR, Jeuland MA, Paul CJ, Belachew DL, McCornick PG. Water Quality Threats, Perceptions of Climate Change and Behavioral Responses among Farmers in the Ethiopian Rift Valley. Climate. 2021;9(6):92.

146. Jamshidi O, Asadi A, Kalantari K, Azadi H, Scheffran J. Vulnerability to climate change of smallholder farmers in the Hamadan province, Iran. Clim Risk Manag. 2019;23:146–59.

147. Evariste FF, Denis Jean S, Victor K, Claudia M. Assessing climate change vulnerability and local adaptation strategies in adjacent communities of the Kribi-Campo coastal ecosystems, South Cameroon. Urban Clim. 2018;24:1037–51.

148. Almudi T, Sinclair AJ. Extreme hydroclimatic events in rural communities of the Brazilian Amazon: local perceptions of change, impacts, and adaptation. Reg Environ Change. 2022;22(1):27.

149. Das S, Mishra AJ. Climate change and the Western Himalayan community: Exploring the local perspective through food choices. Ambio. 2022;12.

150. Olabanji MF, Davis N, Ndarana T, Kuhudzai AG, Mahlobo D. Assessment of smallholder farmers’ perception and adaptation response to climate change in the Olifants catchment, South Africa. Journal of Water and Climate Change. 2021;12(7):3388–403.

151. Chaudhary BR, Acciaioli G, Erskine W, Chaudhary P. Responses of the Tharu to climate change-related hazards in the water sector: Indigenous perceptions, vulnerability and adaptations in the western Tarai of Nepal. Clim Dev. 2021;13(9):816–29.

152. Bacha MS, Nafees M, Adnan S. Farmers Perceptions about Climate Change Vulnerabilities and their Adaptation Measures in District Swat. Sarhad Journal of Agriculture. 2018;34(2).

153. Owusu V, Ma W, Emuah D, Renwick A. Perceptions and vulnerability of farming households to climate change in three agro-ecological zones of Ghana. J Clean Prod. 2021;293:126154.

154. Rehman S, Azhoni A, Chabbi PH. Livelihood vulnerability assessment and climate change perception analysis in Arunachal Pradesh, India. GeoJournal. 2022;21.

155. Dawit M, Dinka MO, Halefom A. Farmers’ perception of climate change and gender sensitive perspective for optimised irrigation in a compound surface-ground water system. Journal of Water and Land Development. 2022;(52):265–71.

156. Sorgho R, Mank I, Kagoné M, Souares A, Danquah I, Sauerborn R. “We Will Always Ask Ourselves the Question of How to Feed the Family”: Subsistence Farmers’ Perceptions on Adaptation to Climate Change in Burkina Faso. Int J Environ Res Public Health. 2020 Oct 1;17(19):7200.

157. Nnadi P, Amadi G. Effects of Climate Change on Tropical Forest Ecosystem of Three Selected Local Government in Rivers State, Nigeria. Journal of Applied Sciences and Environmental Management. 2019;23(1):83.

158. Lumborg S, Tefera S, Munslow B, Mor SM. Examining local perspectives on the influence of climate change on the health of Hamer pastoralists and their livestock in Ethiopia. Pastoralism. 2021;11(1):10.

159. Alfaro AA, Cortés ME. Perception of the impact of climate change on the quality of life and well-being of the inhabitants of the Cerro Blanco Agricultural Community, Limarí Province, Chile. IDESIA (Chile). 2020;38(4):127–31.

160. Ngure MW, Wandiga SO, Olago DO, Oriaso SO. Climate change stressors affecting household food security among Kimandi-Wanyaga smallholder farmers in Murang’a County, Kenya. Open Agric. 2021;6(1):587–608.

161. Obwocha EB, Ramisch JJ, Duguma L, Orero L. The Relationship between Climate Change, Variability, and Food Security: Understanding the Impacts and Building Resilient Food Systems in West Pokot County, Kenya. Sustainability. 2022;14(2):765.

162. Ghosh-Jerath S, Kapoor R, Ghosh U, Singh A, Downs S, Fanzo J. Pathways of Climate Change Impact on Agroforestry, Food Consumption Pattern, and Dietary Diversity Among Indigenous Subsistence Farmers of Sauria Paharia Tribal Community of India: A Mixed Methods Study. Front Sustain Food Syst. 2021;5.

163. Limuwa MM, Synnevåg G. A gendered perspective on the fish value chain, livelihood patterns and coping strategies under climate change - insights from Malawi’s small-scale fisheries. THE AFRICAN JOURNAL OF FOOD, AGRICULTURE, NUTRITION AND DEVELOPMENT. 2018;18(02):13527–46.

164. Kiumbuku SK, Baaru MW, Mutinda JW. Do Smallholder Farmers Perceive Rainfall Variability the Same and Correctly? Gendered and Spatial Analysis of Perception Versus Actual Trends of Rainfall in Three Livelihood Zones in Kenya. Journal of Environment and Earth Science. 2018;8(9):1–12.

165. Diarra FB, Ouédraogo M, Zougmoré RB, Partey ST, Houessionon P, Mensah A. Are perception and adaptation to climate variability and change of cowpea growers in Mali gender differentiated? Environ Dev Sustain. 2021;23(9):13854–70.

166. Addaney M, Sarpong GE, Akudugu JA. Climate Change Adaptation in Akropong, Ghana: Experiences of Female Smallholder Farmers. Journal of Land and Rural Studies. 2021;9(2):344–67.

167. Lawson ET, Alare RS, Salifu ARZ, Thompson-Hall M. Dealing with climate change in semi-arid Ghana: understanding intersectional perceptions and adaptation strategies of women farmers. GeoJournal. 2020;85(2):439–52.
