# Supplementary material for: Existing evidence of conceptual differences in research on climate change perceptions among smallholders? A systematic map
Source: Environ Evid. 2023 Dec 7;12:28. doi: 10.1186/s13750-023-00321-2 (PMC11378835; doi:10.1186/s13750-023-00321-2)
Supplement: Supplementary file 9 — Additional file 9. Information on the theories presented in the articles. [file 13750_2023_321_MOESM9_ESM.docx]

**Additional file 9** Information on the theories presented in the articles (n=361)

| **Name of the theory** | **Field of the theory** | **Description of how the theory was used or cited in the article** | **References** | **Proportion and frequency**  **(% (n))** |
| --- | --- | --- | --- | --- |
| Cognitive biases | Psychology | It argues for the importance of a socio-cognitive approach in decision-making by combining physical data patterns with the experience and viewpoints of individuals. | (1) | 4% (16) |
|  |  | It argues that the social desirability bias likely explains why respondents describe climate change as more problematic during workshops than in interviews. Argues that biases can cause participants to be influenced by the opinions of other participants or of people that supposedly know about the topic (such as scientists, politicians, and others). | (2) |  |
|  |  | It argues that cognition and emotion lead to biases in how people assess risk. | (3) |  |
|  |  | It discusses how intuition and emotion influence decision-making and that cognitive biases are often not considered. | (4) |  |
|  |  | It shows evidence of a cognitive bias related to farmers’ perception of rainfall. | (5) |  |
|  |  | It discusses heuristics as a cognitive bias in decision-making. | (6) |  |
|  |  | The article highlights the delay in considering sociocultural and cognitive systems that shape local assessment of climate change risks and responses. | (7)* |  |
|  |  | It discusses individuals’ difficulties in cognitively understanding the term climate change, its impacts, and adaptation strategies. | (8)(9)* |  |
|  |  | It discusses the importance of combining scientific data with subjective perception data to avoid biases (e.g. heuristic biases). | (10)* (11) |  |
|  |  | It argues that the *status quo* bias explains why some people disregard the effects of climate change. | (12)* |  |
|  |  | It presents the availability heuristic as an example of perceptions not aligned with meteorological data. | (13) |  |
|  |  | It argues that heuristics and biases affect climate change perception. The results present evidence that heuristics in education can affect perceptions, besides presenting evidence of cognitive biases. | (14) |  |
|  |  | It presents psychological barriers to decision-making, such as loss aversion and assimilation biases. | (15) |  |
|  |  | It presents that economic and cognitive factors are determinants of adaptation; therefore, it uses both cognitive and non-cognitive factors in the analyses. | (16)* |  |
| Prospect Theory | Behavioral economics | The theory is adopted to evaluate farmers’ behavior concerning climate change risk perception. | (12)* | 0.55% (2) |
|  |  | The theory is adopted to investigate the effects on people’s climate change perception. It argues that pastoralists are more sensitive to losses due to drought-related climate risks. | (14) |  |
| Protection Motivation Theory | Psychology | It presents the relationship between risk perception and adaptation. Individuals take precautionary measures through two different cognitive processes: threat assessment and coping assessment. | (17)*. | 1% (4) |
|  |  | It argues that cognitive processes motivate individuals through the protection against extreme weather events caused by climate change. | (18)*. |  |
|  |  | The study is conceptualized based on this theory. Thus, when facing risks, an individual’s behavior depends on his/her ability to assess and cope with threats. | (19) |  |
|  |  | It argues that economic and cognitive factors can influence people’s adaptation strategies. | (16)* |  |
| Cultural Theory | Anthropology | It explains how indigenous people understand climate change according to their worldviews, based on the principle that individuals fit into four premises of solidarity: fatalist, individualistic, hierarchical, and egalitarian. | (20) | 0.28% (1) |
| Cognitive models: mental models of climate change | Psychology | It presents how an individual perceive climate change. Mental models are cognitive representations of reality in which an individual’s observation and experience contribute to the representation. | (7)* | 0.28% (1) |
| Climate Change Response Model | Multi-  disciplinary | The model is used to conceptualize climate change and its relation with the resulting vulnerability and impacts. The model ties vulnerability, climate change perception, impacts on smallholder farmers’ livelihoods, and changes in the decision-making process. | (21) | 0.28% (1) |
| Model of Private Proactive Adaptation to climate change | Psychology | The research follows the model developed by Grothmann & Patt (2005)¹ to understand how people assess risks and examine what affects risk perception. Two processes to conceptualize risk perception are presented: risk assessment and adaptation assessment. | (22)*. | 0.55% (2) |
|  |  | It presents a model to argue that economic factors and cognition can affect people’s adaptation strategies. | (16)*. |  |
| Theory of Rational Choice - Expected Utility Theory | Economics | It presents an analytical framework in the methods section, assuming that farmers are rational and choose adaptation strategies to maximize their utility. | (23) | 2% (8) |
|  |  | It argues that the choice of adaptation strategy depends on the utility expected by farmers, besides socioeconomic conditions. The theory is cited in the Methods section (econometric model). | (24) |  |
|  |  | It compares the theory to Prospect Theory. | (12)* |  |
|  |  | It presents an analytical framework implicitly in the Methods, arguing that farmers adhere to new technology when the utility (profit) is greater than that from traditional methods. | (25) |  |
|  |  | It suggests that farmers have enough information about a risk event’s probabilities and impacts/consequences. | (26)*. |  |
|  |  | The theory is presented in the data analysis section (econometric model) to examine what influences adaptation choices, assuming that individuals seek to maximize utility (e.g. higher benefit from the strategy). | (27)*(28) |  |
|  |  | It presents the theory as an example to refer to perceptions misaligned with meteorological data. | (13) |  |
| Random Utility Maximization Theory or Random Utility Model | Economics | The theory describes what the family’s utility function would look like before and after incorporating adaptation strategies. | (29) | 0.80% (3) |
|  |  | It conceptualizes which adaptation strategy would be adopted based on the utility perceived by individuals. | (30,31) |  |
| Integral Theory  (Metatheory) | Multi-  disciplinary | The theory is cited in the Methods section (data collection) to show that the quantitative research preceded the qualitative phase. | (32) | 0.55% (2) |
|  |  | It is argued in the Methods section that the integral theory extracts knowledge from different perspectives to understand environmental issues. | (11) |  |
| Behavioral Decision-Making Theory  (Bounded Rationality Theory) | Economics | In an economic context, it indicates that decision makers are rationally limited and react to threats and opportunities in different ways. It argues that individuals’ short and long-term perceptions of climate change can lead to different adaptive behaviors. | (33)*. | 0.55% (2) |
|  |  | Briefly presents the bounded rationality theory to point out there are cognitive biases. | (14) |  |
| Theory of Planned Behavior | Psychology | It adopts the theory to explore perception and adaptation responses. Three factors were used in the context of climate change: behavioral beliefs (attitude towards behavior), normative beliefs (subjective norms), and control beliefs (behavioral control). When combined, factors give rise to behavioral intention and are linked to the self-efficacy theory). | (34) | 0.55% (2) |
|  |  | It applied the theory to conceptualize rural farmers’ perception and behavior control over climate change and variability, analyzing attitudes towards behavior, subjective norms, and perceived behavioral controls, besides incorporating an adaptation strategy. | (35)* |  |
| Value-Believe-Norm Theory | Psychology | It discusses how beliefs impact the behavior of individuals influenced by psychological factors, such as beliefs, concerns, perceptions, and attitude toward climate change. | (36) | 0.55% (2) |
|  |  | It presents the theory as a framework to analyze individuals’ perceptions and their adaptive behavior toward climate change. Thus, it analyzes trust, climate beliefs, risk perception, and adaptation. | (37)* |  |
| Local theory/explanation based on the grounded theory method. | Social Sciences in general | It explains that theory follows data gathering, i.e. first data was collected and analyzed and only then a theory was developed. | (38) | 0.28% (1) |
| Perceptual Geography Approach | Geography | It argues that individuals’ experiences can affect perception, and suggests that herders’ perception and observation of the environment vary according to different life experiences. | (39) | 0.28% (1) |
| Capital Approach Framework or Capital Theory | Economics | It examines the relationship between farmers’ perceptions and their adaptive assets (human, physical, financial, social, and natural capital) in adaptation strategies. | (40)*. | 1% (4) |
|  |  | It argues that traditional knowledge is incorporated in the theory of social capital. | (41) |  |
|  |  | It examines the impact of climate change in five forms of capitals (social, cultural, natural, physical, and economic) and the use of these capitals in adaptation strategies. | (42) |  |
|  |  | Livelihoods are characterized based on five forms of capital (human, physical, financial, social, and natural). Based on the theory, human, social, natural, physical, and financial characteristics were determined, and seven other variables were considered to assess the perception and willingness to accept mitigation and adaptation actions. | (43)*. |  |
| Norgaard’s theory of denial | Psychology and Sociology | It argues that climate change denial is the inability to integrate knowledge about it into everyday life or transform it into social action. Therefore, poor adaptation would occur due to denial of the problem (even if climate change is recognized, no action is taken) and lack of knowledge. | (15) | 0.28% (1) |

**Note**:* indicates articles that presented an explicit definition of the perception concept; ¹Reference: Grothmann T, Patt A. Adaptive capacity and human cognition: the process of individual adaptation to climate change. Global environmental change. 2005;15(3):199-213.

**References**

1. Bedeke S, Vanhove W, Wordofa M, Natarajan K, van Damme P. Perception of and response to climate change by maize-dependent smallholders. Clim Res. 2018;75(3):261–75.

2. Nef DP, Neneth D, Dini P, Abad CR, Kruetli P. How local communities attribute livelihood vulnerabilities to climate change and other causes: a case study in North Vanuatu. Clim Change. 2021;168(3–4):17.

3. Ensor JE, Abernethy KE, Hoddy ET, Aswani S, Albert S, Vaccaro I, et al. Variation in perception of environmental change in nine Solomon Islands communities: implications for securing fairness in community-based adaptation. Reg Environ Change. 2018;18(4):1131–43.

4. Smith RA. Risk perception and adaptive responses to climate change and climatic variability in northeastern St. Vincent. J Environ Stud Sci. 2018;8(1):73–85.

5. Waldman KB, Vergopolan N, Attari SZ, Sheffield J, Estes LD, Caylor KK, et al. Cognitive biases about climate variability in smallholder farming systems in Zambia. Weather, Climate, and Society. 2019;11(2):369–83.

6. Waldman KB, Attari SZ, Gower DB, Giroux SA, Caylor KK, Evans TP. The salience of climate change in farmer decision-making within smallholder semi-arid agroecosystems. Clim Change. 2019;156(4):527–43.

7. Adaawen S. Understanding Climate Change and Drought Perceptions, Impact and Responses in the Rural Savannah, West Africa. Atmosphere (Basel). 2021;12(5):594.

8. Shijin W. Perception of indigenous people of climate change and its impact on the Everest National Nature Preserve. Meteorological Applications. 2021;1–12.

9. Nnko HJ, Gwakisa PS, Ngonyoka A, Estes A. Climate change and variability perceptions and adaptations of pastoralists’ communities in the Maasai Steppe, Tanzania. J Arid Environ. 2021;185:104337.

10. Behailu G, Ayal DY, Zeleke TT, Ture K, Bantider A. Comparative Analysis of Meteorological Records of Climate Variability and Farmers’ Perceptions in Sekota Woreda, Ethiopia. Clim Serv. 2021;23:100239.

11. Guodaar L, Bardsley DK, Suh J. Integrating local perceptions with scientific evidence to understand climate change variability in northern Ghana: A mixed-methods approach. Applied Geography. 2021;130:102440.

12. Villacis AH, Alwang JR, Barrera V. Linking risk preferences and risk perceptions of climate change: A prospect theory approach. Agricultural Economics. 2021;52(5):863–77.

13. Kannan S, Bessette DL, Abidoye B. Misalignment of perceptions with records and resources for responding to climate change risk. Frontiers in Climate. 2022;4.

14. Nkuba MR, Chanda R, Mmopelwa G, Kato E, Najjingo Mangheni M, Lesolle D, et al. Effect of indigenous and scientific forecasts on pastoralists’ climate change perceptions in the Rwenzori region, Western Uganda. Clim Dev. 2022;1–13.

15. Sharma R, Jagtap S, Rao P. Understanding Maharashtra Coastal Community’s Perceptions and Livelihood Resilience to Climate Change Using the Community Participatory Approach. The International Journal of Climate Change: Impacts and Responses. 2022;14(2):1–19.

16. Koirala P, Kotani K, Managi S. How do farm size and perceptions matter for farmers’ adaptation responses to climate change in a developing country? Evidence from Nepal. Econ Anal Policy. 2022;74:188–204.

17. Budhathoki NK, Paton D, A. Lassa J, Zander KK. Assessing farmers’ preparedness to cope with the impacts of multiple climate change-related hazards in the Terai lowlands of Nepal. International Journal of Disaster Risk Reduction. 2020;49:101656.

18. Budhathoki NK, Paton D, Lassa JA, Bhatta GD, Zander KK. Heat, cold, and floods: exploring farmers’ motivations to adapt to extreme weather events in the Terai region of Nepal. Natural Hazards. 2020;103(3):3213–37.

19. Poudyal NC, Joshi O, Hodges DG, Bhandari H, Bhattarai P. Climate change, risk perception, and protection motivation among high-altitude residents of the Mt. Everest region in Nepal. Ambio. 2021;50(2):505–18.

20. Ambrosio-Albala DrP, Mar Delgado-Serrano DrM. Understanding Climate Change Perception in Community-Based Management Contexts: Perspectives of Two Indigenous Communities. Weather, Climate, and Society. 2018;10(3):471–85.

21. Maliki MA, Pauline NM. Living and Responding to Climatic Stresses: Perspectives from Smallholder Farmers in Hanang’ District, Tanzania. Environ Manage. 2022;14.

22. Sherpa SF, Shrestha M, Eakin H, Boone CG. Cryospheric hazards and risk perceptions in the Sagarmatha (Mt. Everest) National Park and Buffer Zone, Nepal. Natural Hazards. 2019;96(2):607–26.

23. Addis Y, Abirdew S. Smallholder farmers’ perception of climate change and adaptation strategy choices in Central Ethiopia. Int J Clim Chang Strateg Manag. 2021;13(4/5):463–82.

24. Ali E. Farm Households’ Adoption of Climate-smart Practices in Subsistence Agriculture: Evidence from Northern Togo. Environ Manage. 2021;67(5):949–62.

25. Singh NP, Anand B, Srivastava SK, Kumar NR, Sharma S, Bal SK, et al. Risk, perception and adaptation to climate change: evidence from arid region, India. Natural Hazards. 2022;112(2):1015–37.

26. Raihan ML, Basu M, Onitsuka K, Hoshino S. Determinants of Farmers’ Risk Perceptions of Hailstorms in Northern Bangladesh: Is Adaptive Capacity the Major Concern? Pol J Environ Stud. 2021;31(1):257–70.

27. Mamun A Al, Roy S, Islam ARMdT, Alam GMM, Alam E, Chandra Pal S, et al. Smallholder Farmers’ Perceived Climate-Related Risk, Impact, and Their Choices of Sustainable Adaptation Strategies. Sustainability. 2021;13(21):11922.

28. Megersa GG, Jaleta M, Tesfaye K, Getnet M, Tana T, Lakew B. Perceived Climate Change and Determinants of Adaptation Responses by Smallholder Farmers in Central Ethiopia. Sustainability. 2022;14(11):6590.

29. Mutandwa E, Hanyani-Mlambo B, Manzvera J. Exploring the link between climate change perceptions and adaptation strategies among smallholder farmers in Chimanimani district of Zimbabwe. Int J Soc Econ. 2019;46(7):850–60.

30. Wale E, Nkoana MA, Mkuna E. Climate change-induced livelihood adaptive strategies and perceptions of forest-dependent communities: The case of Inanda, KwaZulu-Natal, South Africa. Trees, Forests and People. 2022;8:100250.

31. Ayansa AD, Bedemo A, Jara GO. Smallholder Farmers‟ Perception and Adaptation to Climate Change in  Kurmuk District, Ethiopia. Applied Economics and Finance. 2021;8(5):29–36.

32. Guodaar L, Bardsley DK, Suh J. Indigenous adaptation to climate change risks in northern Ghana. Clim Change. 2021 May 27;166(1–2):24.

33. Yang S, Yu L, Leng G, Qiu H. Livestock farmers’ perception and adaptation to climate change: panel evidence from pastoral areas in China. Clim Change. 2021;164(1–2):21.

34. Jellason N, Baines R, Conway J, Ogbaga C. Climate Change Perceptions and Attitudes to Smallholder Adaptation in Northwestern Nigerian Drylands. Soc Sci. 2019;8(2):31.

35. Belay A, Oludhe C, Mirzabaev A, Recha JW, Berhane Z, Osano PM, et al. Knowledge of climate change and adaptation by smallholder farmers: evidence from southern Ethiopia. Heliyon. 2022;8(12):e12089.

36. Tiet T, To-The N, Nguyen-Anh T. Farmers’ behaviors and attitudes toward climate change adaptation: evidence from Vietnamese smallholder farmers. Environ Dev Sustain. 2022;26.

37. Peng Y, Xu Z, Wei P, Cheng L. Smallholder farmers’ behavioral preferences under the impact of climate change: A comparative analysis of two agricultural areas in China. Front Earth Sci (Lausanne). 2022;10.

38. Mugambiwa SS, Rukema JR. Climate Change and Variability Discourse among Community Members and Smallholder Farmers in Mutoko  District, Zimbabwe. Mankind Quartely . 2020;61:225–50.

39. Rasmus S, Wallen H, Turunen M, Landauer M, Tahkola J, Jokinen M, et al. Land-use and climate related drivers of change in the reindeer management system in Finland: Geography of perceptions. Applied Geography. 2021;134:102501.

40. Nguyen-Anh T, Nong D, Leu S, To-The N. Changes in the environment from perspectives of small-scale farmers in remote Vietnam. Reg Environ Change. 2021;21(4):98.

41. Maliao RJ, Cahilig RC, Cahilig RR, Jaspe BT. Climate Change Awareness and Indigenous Knowledge Systems and Practices (Iksp) of Riverine Fishers in the Nabaoy River, Malay, Aklan, Philippines: Linking Local Social Capital to Socio-Ecological Resilience Amidst the Changing Environment and Climate. SSRN Electronic Journal. 2022.

42. Guáqueta-Solórzano VE, Postigo JC. Indigenous perceptions and adaptive responses to the impacts of climate variability in the Sierra Nevada de Santa Marta, Colombia. Frontiers in Climate. 2022;4.

43. Torres B, Cayambe J, Paz S, Ayerve K, Heredia-R M, Torres E, et al. Livelihood Capitals, Income Inequality, and the Perception of Climate Change: A Case Study of Small-Scale Cattle Farmers in the Ecuadorian Andes. Sustainability. 2022;14(9):5028.
