# Supplementary material for: Existing evidence of conceptual differences in research on climate change perceptions among smallholders? A systematic map
Source: Environ Evid. 2023 Dec 7;12:28. doi: 10.1186/s13750-023-00321-2 (PMC11378835; doi:10.1186/s13750-023-00321-2)
Supplement: Supplementary file 10 — Additional file 10. Information about constructs (n = 361). [file 13750_2023_321_MOESM10_ESM.docx]

**Additional file 10** Information about constructs (n=361)

| **Name and description of the construct** | **Examples of how it appears in the article** | **Proportion and frequency (% (n))** |
| --- | --- | --- |
| Direct exposure: experiencing, observing, or having direct contact with the effects of climate change, such as heat waves, changes in seasons, warmer summers or winters, less rain, milder winters, less snow, and changes in animal and plant species (1). | *“Perceptions are also associated with experience, i.e., how individuals react to situations.”* (2, p.3) | 99% (359) |
|  | *“In Table 5, smallholder farmers’ experiences of climate change are presented. The majority of the farmers have experienced late onset and early cessation of rainfall in the district as reported by 82.8% and 89.2% of respondents, respectively. (...) A vast majority of the smallholder farmers (94.7%) have also experienced decrease in the duration of rainfall while about 82.5% of the farmers have observed an increase in rainfall intensity.”* (3, p.6) |  |
|  | *“Disaggregated results show that perception varies with the region. A majority of respondents who had experienced most of the considered shocks came from the hill region (e.g., drought, untimely rain, irregular weather, hailstorm, etc.).”* (4, p.9) |  |
|  | *“In this study, farmers’ perception of climate variability was defined by their experiences during the decade which preceded the survey in Tharaka-Nithi County (2007-2017) regarding seven climatic characteristics and several consequences that they had experienced as a result of climate variability. These indicators included change in temperature, change in rainfall amounts, change in rainfall onset and rainfall cessation dates, change in length of the cropping season, and changes in flooding and drought frequency.”* (5, p.2) |  |
| Perception: “the set of processes by which we recognize, organize, and make sense of the sensations we receive from environmental stimuli” (6, p.535). Perception includes sensory experience, including sight (observation), hearing, touch, taste, smell, and other stimuli received from the environment. People interpret stimuli as meaningful based on prior experiences, knowledge, and subjective interpretations of reality (40, 42). | *“Majority of the respondents 72.5% in Dawu village were having a personal experience to judge the climate change issue while only 30% in Daiqian village have examined the climate change through personal observations (Table 7).”* (8. p.7998) | 96% (345) |
|  | *“From Table 3, majority of the respondents (95.9%) had observed at least a change in one element of climate before while the remaining 4.1% had not observed any changes at all. (...) Majority of the respondents mentioned protracted drought, unpredictable rainfall pattern, high temperatures, strong winds and frequent flood events as observed changes in the climate, which negatively affects their agricultural venture.”*(9, p.6) |  |
|  | *“Table 4 captures the respondents’ perceptions of climate change in terms of observed changes in ecological processes.”* (10, p.8) |  |
|  | *“Most of the respondents (84.12%) felt the increase in temperature and the number of hot days."* (11, p.8) |  |
| Awareness: in the context of climate change, it's “the process to perceive and understand CC as a hazard as well as to increase the willingness of taking action in a committed and collaborative way to adapt and face the challenges of climate change” (12, p.3). Awareness of climate change includes perception of climate change as a problem and a threat (13). | *“The extent to which a community is aware of climate change reflects its level of exposure to climate risks. The study reveals that 84.4% of the respondents were aware of climate change whereas 15.6% were not. Regarding livelihood strategies, farmers recorded the highest score of awareness (87.5%), followed by agropastoralists (78%). The level of awareness among Fulani people (92.9%) was higher than that of Hausa people (83.60%). In terms of gender, women (66.7%) were less aware than men (80.5%).”*(14, p.2969-2970) | 53% (191) |
|  | *“As it is indicated in the Table 1, the majorities (90.3%) of the respondents reported that they have awareness about climate change in their communities while the remaining 9.7% of them have never come across and aware of the phrase climate change in their local communities.”* (15, p.4) |  |
|  | *“The data presented in Figure 5 has shown the responses of the pastoral communities about climate change perception. Responding to the question whether they know about climate change or not. 83.3% of the respondents belonging to Daiqian village were aware of the climate change issues while about 16.7% of the respondents were not aware of the climate change phenomenon at all. As compared to Daiqian, the pastoral community of the Dawu was less aware of the climate change. However majority of the respondents of Dawu village75% had the knowledge about climate change while 25% did not have awareness about climate change (Table 6).”* (8, p.7997-7998) |  |
|  | *“In the savannah, all the respondents (100%) were aware of changes in rainfall pattern and intensity. Nearly all of them (95.5%) were aware of changes in temperature and 78.5% of changes in heat levels. Similarly, in the rainforest, a large proportion of the respondents (92.5%) were aware of changes in rainfall patterns and 78.5% of changes in rainfall intensity. The majority were aware of changes in temperature (87.5%) and 67.5% of changes in heat levels. The high level of awareness of the indicators is connected with the temperature fluctuation observed from the trend analysis.”* (16, p.8) |  |
| Belief: “a personal conviction that is not necessarily supported by science-based evidence - but that is shaped by the overall context in which they occur, including the scientific understanding we have of it.” (17, p.2). Aspects learned through experience or school, or even misconceptions and even incomplete truths that people consider to be valid beliefs (18). | *“People in the study area exhibit different perceptions on the causes of climate change. Although they were unanimous on the multiple causes of climate change, they however, differ in their ranking of these causes based on their degree of influence in changing the climate of the area. Figure 7 above, depicts this divergence of opinions. 39% of the respondents regarded climate change as something naturally caused by God.”* (19, p.75) | 35% (125) |
|  | *“About 83% of the farmers were not aware of the term “climate change,” yet the majority of the farmers (97%) believed that climate has certainly changed from what they recall of 20–25 years ago.”* (20, p.111) |  |
|  | *“Perception is also about the beliefs an individual or a group have about an issue.”*(21, p.3) |  |
|  | *“In discussing the causes of CC, the farmers’ responses aligned under two categories: CC is due to “Godly Actions” and/ or “Human Actions.””* (22, p.5) |  |
| Indirect exposure: The individual is physically distant from a climate change situation and may hear, read, or see the phenomenon in another person or place (23). Indirect experience may include: (i) observing other individuals and feeling what they feel through second- or third-hand reports of known or unknown people (vicarious experience) (24), and (ii) experiencing the phenomenon through media coverage, such as newspapers, television, and the internet (virtual experience). | *“The results from the analysis also show that information from family and friends, and government significantly influence farmers' perception.”* (3, p.9) | 28% (101) |
|  | *“The other factor contributing in the development of climate change awareness among pastoralist included Government Departments, weather forecast program, TV programs and multiple sources. Weather forecast program and TV program jointly contributed in developing perception among 13 (43%) and 16 (40%) of the respondents in Daiqian and Dawu respectively.”*(8, p.7998) |  |
|  | *“Of the farmers that were aware of the term, climate change have acquired knowledge through camps mostly arranged by local NGOs.”* (20, p.112) |  |
|  | *“All farmers, except one, were introduced to CC through one of four sources: local radio show, informal discussions, provincial service agents, and their own experiences.”* (22, p.5) |  |
| Traditional knowledge: “The knowledge, innovations, and practices of both indigenous and local communities around the world that are deeply grounded in history and experience” (25, p.1774). Therefore, a knowledge that: (i) tends to be the result of cumulative experiences and observations tested in the context of everyday life; (ii) passed through oral communication and repetitive engagement, and not through formal instruction; (iii) dynamic and adapted to cultural and environmental changes, and (iv) generally transmitted orally from one generation to another (25) | *“This means that they still rely significantly on their experiences based on personal physical observation of the environment. These ways of observing their natural world and harmonious ways of existing within this world has been passed along from generation to generation throughout time using an oral tradition. For weather forecasting, local people have developed personal techniques involving the keen observation of various faunal and floral movements, as well as being uniquely attuned to other physical changes in their surroundings, recorded in Table 3.”* (26, p.8) | 23% (82) |
|  | *“During focus group discussions, it was evident that farmers in Nwanedi and Levubu have historically utilized a number of indigenous indicators for weather forecasts based on socio-cultural and environmental beliefs, but with limited documentation. During the field survey, major indicators employed by farmers for forecasting weather and climate change were analyzed.”* (27, p.9) |  |
|  | *“Indigenous knowledge about climate change refers to understandings and philosophies developed by long-established nomad communities on climate change, and this knowledge has a strong link with the natural environment. (...) Thus, indigenous knowledge is referred to as traditional knowledge or local ecological perceptions, which has received much attention in climate change discussions and adaptation mechanisms.”* (28, p.16745) |  |
|  | *“Information obtained during the focus group discussion and interviews with key informants from both villages affirmed that farmers often use their local knowledge for weather predictions in helping them to take appropriate actions in response to an anticipated worse situation.”* (29, p.7) |  |
| Concern: a feeling of concern about the results and consequences of climate change (30) | *“Whether people are concerned about climate change is a basic construct often attempted to explore in repeated national and international surveys. The current survey attempted to measure this important construct by asking farmers how much worried they are (instead of how much concerned they are) about climate change and its impacts.”* (31, p.1748) | 20% (74) |
|  | *“Almost all the fishermen who participated in the study expressed concern and fear about the increasing intensity of climate events and the resulting livelihoods insecurity over the years.”* (32, p.3474) |  |
|  | *“It is notable that Kashkoolis’ awareness of climate change had become strong, although concern has been variable among households.”* (28, p.16762) |  |
|  | *“To facilitate dialog with informants during interviews about perceived risks from climate change, expressions such as “problems,” “concerns,” and “bad situations” were used as synonyms for “risk.””* (33, p.406) |  |
| Risk perception: “The subjective judgment that people make about the characteristics and severity of a risk” (25, p.1772). In this case, the risks associated with climate change. The risk is often represented as the “probability of occurrence of hazardous events or trends multiplied by the impacts if these events or trends occur. Risk results from the interaction of vulnerability, exposure, and hazard.” (25, p.1772) | *“In this study, we introduced the Climate Change Risk Perception Index (CCRPI) for measuring char dwellers’ climate change risk perception against any climatic events based on their personal experience.”* (34, p.3) | 12% (45) |
|  | *“Risk perceptions about the climate consequences (i.e., perceived risk) often link to farmers’ awareness about whether climate change will affect their farming activities in the future or not “* (35, p.4) |  |
|  | *“In this study, we used the climate change risk perception index (CCRPI) for the calculation of livestock herders’ perceptions of climatic events/variations that occurred during the past 10 years but rarely occurred previously.”* (36, p.5) |  |
|  | *“The perception of risk is, therefore, a mental construct (Sjöberg, 2000) that distinguishes between the existence of objective real-world threats and the subjective evaluation of those threats. The subjective evaluation of threats can make climate change risk perceptions vary significantly among individuals.”* (37, p.864) |  |
| Attitude: “a psychological tendency that is expressed by evaluating a particular entity with some degree of favor or disfavor” (38, p.1). “Attitudes have a subject matter, which can be an object, a person, or an abstract idea”(39, p.300) | *“An understanding of the current level of knowledge, attitudes, and perceptions of farmers serve as an indicator of how well informed they are about climate change and how well-positioned they are to adopt climate change adaptation methods.”* (40, p.320) | 5% (17) |
|  | *“Climate change awareness involves creating knowledge, understanding and values, attitude, skills, and abilities among individuals and social groups towards the issues of climate change for attaining a better-quality environment.”* (41, p.240) |  |
|  | *“Essentially, climate change and extreme events perception are complex processes that encompass a range of psychological constructs, such as knowledge, beliefs, attitudes, and concerns about whether and how the climate is changing.”* (42, p.3) |  |
|  | *“It is fundamental to consider that the perception of climate change is a complex process that encompasses a variety of psychological constructs, such as the knowledge, beliefs, attitudes, and concerns about whether and how the climate is changing.”* (43, p;13) |  |
| Scientific Knowledge: “usually referring to the often explicit knowledge that has been derived from applying more formal methods that aim to increase rigor in relation to different positions on validity and reliability. This includes natural science and social science research” (44, p.1769) | *“Perceptions of these changes by rural communities are concentrated on observations of variations in temperature, rainfall, and vegetation patterns, which are often backed by blending such perceptions with scientific evidence.”* (45, p.2) | 2% (8) |
|  | *“The colonial heritage and the influence of Catholicism in the Tequendama region (where this municipality is located) may explain the prevalence of religious aspects in the way of knowing and explaining the world and climate phenomena. This is also related to the limited access to scientific information, since only 3% of the participants have received any kind of training in the subject.”* (46, p.11) |  |
|  | *“At the beginning of each participatory community workshop, the fisheries and biodiversity group analyzed and evaluated the potential impacts of climate change on fishing activities and biodiversity, taking into account the available scientific evidence for climate change effects in the region (e.g. the information published in the Intergovernmental Panel on Climate Change’s Fifth Assessment Report).”* (47, p.3) |  |
|  | *“As the Tharu have not had access to any specific flooding prediction technologies, they have relied on their IK, as well as scientific weather forecasts. Tharu IK uses a combination of physical and biological indicators to predict extreme climate phenomena, such as excessive rainfall and droughts. (...) The Tharu have embraced ‘hybrid knowledge’ – a combination of Indigenous and scientific knowledge, technology and practice to increase yield and maximize profit as well as decrease vulnerability to extreme weather events.”* (48, p.8) |  |
| Worldview: implies a wide and coherent set of values related to a particular aspect of life (49), in this case, climate change. Consider worldview as people's set of values that organizes and integrates what they learned about the world and themselves (e.g. through experience) in a symbolic representation system related to their views on climate change (50) | *“The perceptions that emerged were interpreted according to the four worldviews proposed by cultural theory.”* (51. p.471) | 2% (7) |
|  | *“How people perceive (in the broader sense of understand or interpret) climatic changes is shaped by their broader concerns and priorities and how they identify with particular groups. It is also founded on the ontological and epistemological underpinnings of their worldviews, which may differ significantly from the dominant understanding upheld by climate science and upon which mainstream responses to climate change are singularly based.”* (52, p.346) |  |
|  | *“Perception of climate change hazards can vary between members of different ethnic groups due to different perceptions or world views.”* (53, p.3) |  |
|  | *“The perception of the ecological system varies according to the worldview of each social system or ethnic group.* (54, p.93) |  |

**References**

1. Akerlof K, Maibach EW, Fitzgerald D, Cedeno AY, Neuman A. Do people “personally experience” global warming, and if so how, and does it matter? Global Environmental Change. 2013;23(1):81–91.

2. Limuwa M, Sitaula B, Njaya F, Storebakken T. Evaluation of Small-Scale Fishers’ Perceptions on Climate Change and Their Coping Strategies: Insights from Lake Malawi. Climate. 2018;6(2):34.

3. Asare-Nuamah P, Botchway E. Comparing smallholder farmers’ climate change perception with climate data: the case of Adansi North District of Ghana. Heliyon. 2019;5(12):e03065.

4. Pandey VP, Sharma A, Dhaubanjar S, Bharati L, Joshi IR. Climate Shocks and Responses in Karnali-Mahakali Basins, Western Nepal. Climate. 2019;7(7):92.

5. Mairura FS, Musafiri CM, Kiboi MN, Macharia JM, Ng’etich OK, Shisanya CA, et al. Determinants of farmers’ perceptions of climate variability, mitigation, and adaptation strategies in the central highlands of Kenya. Weather Clim Extrem. 2021;34:1–14.

6. Sternberg, Robert J.; Sternberg, Karin; Mio Jeff. Cognitive Psychology. Cognitive Psychology. Wadsworth, Cengage Learning; 2012.

7. Pike G, Edgar G, Edgar H. Perception. In: Braisby N, Gellatly A, editors. Cognitive psychology. Oxford University Press; 2012. p. 65–99.

8. Sharif J, Rafiq MK, Rafiq MT, Aziz R, Qayyum A, Saleem AR, et al. Climate change perceptions and adaptative actions by pastoral community of the Tibetan Plateau, China. Appl Ecol Environ Res. 2019;17(4).

9. Asante F, Guodaar L, Arimiyaw S. Climate change and variability awareness and livelihood adaptive strategies among smallholder farmers in semi-arid northern Ghana. Environ Dev. 2021;39:100629.

10. Chanza N, Musakwa W. Ecological and Hydrological Indicators of Climate Change Observed by Dryland Communities of Malipati in Chiredzi, Zimbabwe. Diversity (Basel). 2022;14(7):541.

11. Rai S, Dahal B, Anup KC. Climate change perceptions and adaptations by indigenous Chepang community of Dhading, Nepal. GeoJournal. 2022;16.

12. Iturriza M, Labaka L, Hernantes J, Abdelgawad A. Shifting to climate change aware cities to facilitate the city resilience implementation. Cities. 2020;101:102688.

13. Arlt D, Hoppe I, Wolling J. Climate change and media usage: Effects on problem awareness and behavioural intentions. Int Commun Gaz. 2011;73(1–2):45–63.

14. Ado AM, Leshan J, Savadogo P, Bo L, Shah AA. Farmers’ awareness and perception of climate change impacts: case study of Aguie district in Niger. Environ Dev Sustain. 2019;21(6):2963–77.

15. Hundera H, Mpandeli S, Bantider A. Smallholder farmers’ awareness and perceptions of climate change in Adama district, central rift valley of Ethiopia. Weather Clim Extrem. 2019;26:100230.

16. Akano O, Modirwa S, Oluwasemire K, Oladele O. Awareness and perception of climate change by smallholder farmers in two agroecological zones of Oyo state Southwest Nigeria. GeoJournal. 2022;30.

17. Sousa-Silva R, Ponette Q, Verheyen K, van Herzele A, Muys B. Adaptation of forest management to climate change as perceived by forest owners and managers in Belgium. For Ecosyst. 2016;3(1):22.

18. Ardoin N, Heimlich J, Braus J, Merrick C. Influencing Conservation Action: What Research Says about Environmental Literacy. Behavior, and Conservation Results; 2013.

19. Jibrillah AM, Jaafara M, Choy LK. Climate Change Awareness and Adaptations Among the Farming and Animal Rearing Communities of the Central Sokoto Close-Settle Zone, North-Western Nigeria. Jurnal Kejuruteraan SI. 2018;1(6):71–9.

20. Shukla R, Agarwal A, Sachdeva K, Kurths J, Joshi PK. Climate change perception: an analysis of climate change and risk perceptions among farmer types of Indian Western Himalayas. Clim Change. 2019;152(1):103–19.

21. Sraku-Lartey M, Buor D, Adjei POW, Foli EG. Perceptions and knowledge on climate change in local communities in the Offinso Municipality, Ghana. Information Development. 2020;36(1):16–35.

22. Sorgho R, Mank I, Kagoné M, Souares A, Danquah I, Sauerborn R. “We Will Always Ask Ourselves the Question of How to Feed the Family”: Subsistence Farmers’ Perceptions on Adaptation to Climate Change in Burkina Faso. Int J Environ Res Public Health. 2020;17(19):7200.

23. Hamilton-Webb A, Manning L, Naylor R, Conway J. The relationship between risk experience and risk response: a study of farmers and climate change. J Risk Res. 2017;20(11):1379–93.

24. Paton D, Johnston DM, Bebbington M, Lai CD. Direct and vicarious experience of volcanic hazards: implications for risk perception and adjustment adoption. . he Australian Journal of Emergency Management. 2000;15(4):58–63.

25. IPCC. Annex II Glossary. WGIIAR5. Agard J, Schipper ELF, Birkmann J, Campos M, Dubeux C, Nojiri Y, et al., editors. IPCC; 2014. 1757–1776 p.

26. van Huynh C, Phuong Le QN, Hong Nguyen MT, Tran PT, Nguyen TQ, Pham TG, et al. Indigenous knowledge in relation to climate change: adaptation practices used by the Xo Dang people of central Vietnam. Heliyon. 2020;6(12):e05656.

27. Kom Z, Nethengwe NS, Mpandeli S, Chikoore H. Indigenous knowledge indicators employed by farmers for adaptation to climate change in rural South Africa. Journal of Environmental Planning and Management. 2022;1–16.

28. Ghazali S, Azadi H, Janeckova K, Sklenicka P, Kurban A, Cakir S. Indigenous knowledge about climate change and sustainability of nomadic livelihoods: understanding adaptability coping strategies. Environ Dev Sustain. 2021;23(11):16744–68.

29. Maliki MA, Pauline NM. Living and Responding to Climatic Stresses: Perspectives from Smallholder Farmers in Hanang’ District, Tanzania. Environ Manage. 2022;14.

30. Poortinga W, Whitmarsh L, Steg L, Böhm G, Fisher S. Climate change perceptions and their individual-level determinants: A cross-European analysis. Global Environmental Change. 2019;55:25–35.

31. Lone FA, Maheen M, ul Shafiq M, Bhat MS, Rather JA. Farmer’s perception and adaptation strategies to changing climate in Kashmir Himalayas, India. GeoJournal. 2022;87(3):1743–57.

32. Madhanagopal D, Pattanaik S. Exploring fishermen’s local knowledge and perceptions in the face of climate change: the case of coastal Tamil Nadu, India. Environ Dev Sustain. 2020;22(4):3461–89.

33. Magalhães HF, Feitosa IS, de Lima Araújo E, Albuquerque UP. Perceptions of Risks Related to Climate Change in Agroecosystems in a Semi-arid Region of Brazil. Hum Ecol. 2021;49(4):403–13.

34. Ahmed Z, Guha GS, Shew AM, Alam GMM. Climate change risk perceptions and agricultural adaptation strategies in vulnerable riverine char islands of Bangladesh. Land use policy. 2021;103:105295.

35. Tiet T, To-The N, Nguyen-Anh T. Farmers’ behaviors and attitudes toward climate change adaptation: evidence from Vietnamese smallholder farmers. Environ Dev Sustain. 2022;26.

36. Faisal M, Abbas A, Cai Y, Ali A, Shahzad MA, Akhtar S, et al. Perceptions, Vulnerability and Adaptation Strategies for Mitigating Climate Change Effects among Small Livestock Herders in Punjab, Pakistan. Int J Environ Res Public Health. 2021;18(20):10771.

37. Villacis AH, Alwang JR, Barrera V. Linking risk preferences and risk perceptions of climate change: A prospect theory approach. Agricultural Economics. 2021;52(5):863–77.

38. Chaiken S, Eagly AH. The psychology of attitudes. Cengage Learning. 1993;1–21.

39. Albarracin D, Shavitt S. Attitudes and Attitude Change. Annu Rev Psychol. 2018;69(1):299–327.

40. Ayanlade A, Radeny M, Akin-Onigbinde AI. Climate variability/change and attitude to adaptation technologies: a pilot study among selected rural farmers’ communities in Nigeria. GeoJournal. 2018;83(2):319–31.

41. Sen LTH, Bond J, Phuong LTH, Winkel A, Tran UC, Le N van. The importance of climate change awareness for the adaptive capacity of ethnic minority farmers in the mountainous areas of Thua Thien Hue province. Local Environ. 2021;26(2):239–51.

42. Damtew A, Teferi E, Ongoma V, Mumo R, Esayas B. Spatiotemporal Changes in Mean and Extreme Climate: Farmers’ Perception and Its Agricultural Implications in Awash River Basin, Ethiopia. Climate. 2022;10(6):89.

43. Torres B, Cayambe J, Paz S, Ayerve K, Heredia-R M, Torres E, et al. Livelihood Capitals, Income Inequality, and the Perception of Climate Change: A Case Study of Small-Scale Cattle Farmers in the Ecuadorian Andes. Sustainability. 2022;14(9):5028.

44. Raymond CM, Fazey I, Reed MS, Stringer LC, Robinson GM, Evely AC. Integrating local and scientific knowledge for environmental management. J Environ Manage. 2010;91(8):1766–77.

45. Tume SJP, Kimengsi JN, Fogwe ZN. Indigenous Knowledge and Farmer Perceptions of Climate and Ecological Changes in the Bamenda Highlands of Cameroon: Insights from the Bui Plateau. Climate. 2019;7(12):138.

46. Córdoba Vargas CA, Hortúa Romero S, León-Sicard T. Resilience to climate variability: the role of perceptions and traditional knowledge in the Colombian Andes. Agroecology and Sustainable Food Systems. 2020;44(4):419–45.

47. Salvadeo C, Morzaria-Luna HN, Reyes-Bonilla H, Ivanova-Bonchera A, Ramírez DP, Juárez-León E. Fisher’s perceptions inform adaptation measures to reduce vulnerability to climate change in a Mexican natural protected area. Mar Policy. 2021;134:104793.

48. Chaudhary BR, Acciaioli G, Erskine W, Chaudhary P. Responses of the Tharu to climate change-related hazards in the water sector: Indigenous perceptions, vulnerability and adaptations in the western Tarai of Nepal. Clim Dev. 2021;13(9):816–29.

49. Kalberg S. The Past and Present Influence of World Views. Journal of Classical Sociology. 2004;4(2):139–63.

50. Aerts D, van Belle H, van der Veken J. World Views and the Problem of Synthesis: The Yellow Book of “Einstein Meets Magritte”. Springer Science & Business Media; 2012.

51. Ambrosio-Albala DrP, Mar Delgado-Serrano DrM. Understanding Climate Change Perception in Community-Based Management Contexts: Perspectives of Two Indigenous Communities. Weather, Climate, and Society. 2018;10(3):471–85.

52. Scoville-Simonds M. Climate, the Earth, and God – Entangled narratives of cultural and climatic change in the Peruvian Andes. World Dev. 2018;110:345–59.

53. Quandt A. Variability in perceptions of household livelihood resilience and drought at the intersection of gender and ethnicity. Clim Change. 2019;152(1):1–15.

54. Ramírez KDI, Ibarra AMA. Less Rain and More Heat”: Smallholders’ Perception and Climate Change Adaptation Strategies in Tropical Environments. Sociedad y Ambiente. 2019;(21):77–104.
