# Supplementary material for: Existing evidence of conceptual differences in research on climate change perceptions among smallholders? A systematic map
Source: Environ Evid. 2023 Dec 7;12:28. doi: 10.1186/s13750-023-00321-2 (PMC11378835; doi:10.1186/s13750-023-00321-2)
Supplement: Supplementary file 11 — Additional file 11. Constructs by geographical distribution. [file 13750_2023_321_MOESM11_ESM.docx]

**Additional file 11** Constructs by geographical distribution (n=361)

| 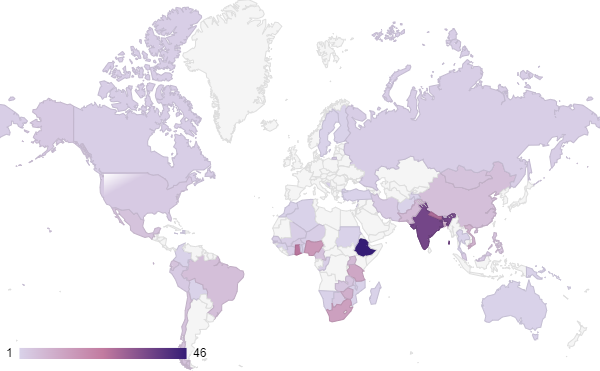  (a) Perception | 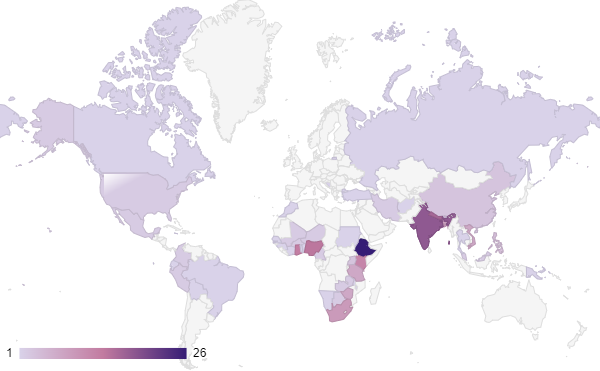  (b) Awareness |
| --- | --- |
| 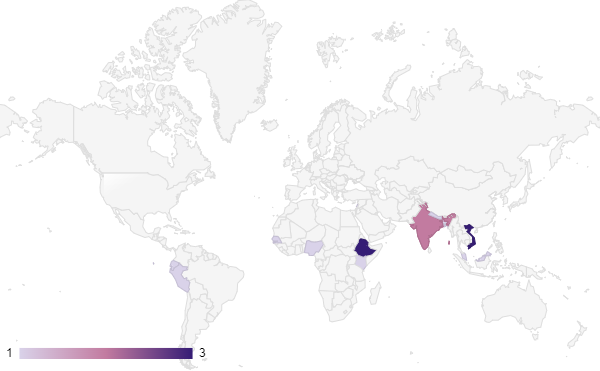  (c) Attitude | 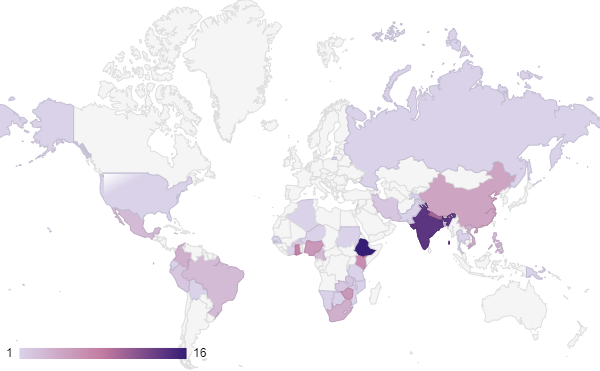  (d) Belief |
| 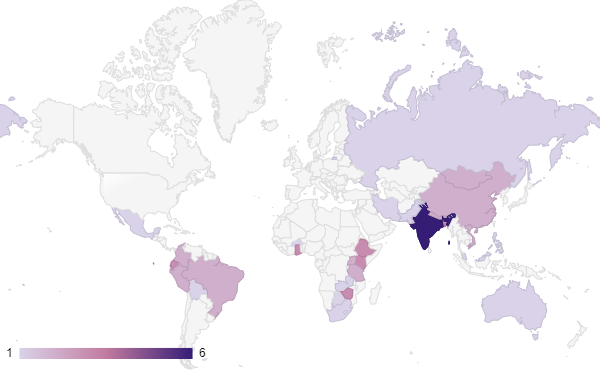  (e) Concern | 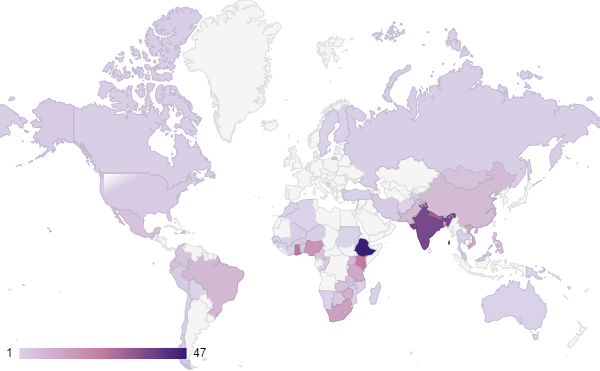  (f) Direct experience |
| 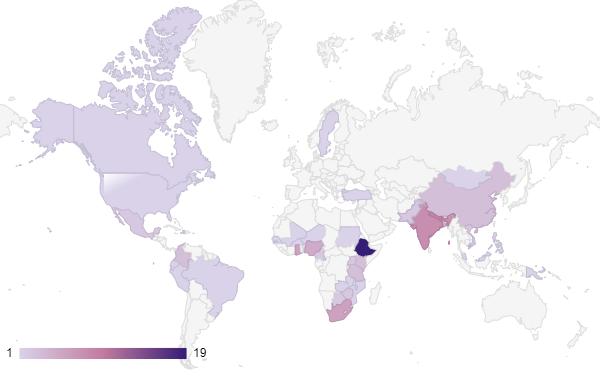  (g) Indirect experience | 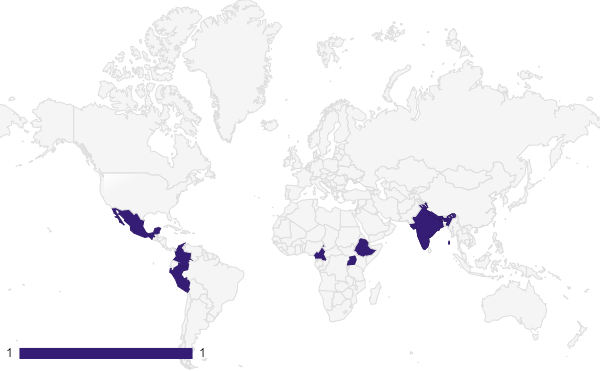  (h) Scientific knowledge |
| 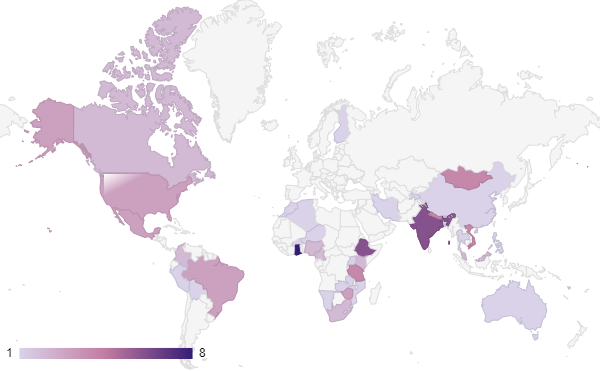  (i) Traditional knowledge | 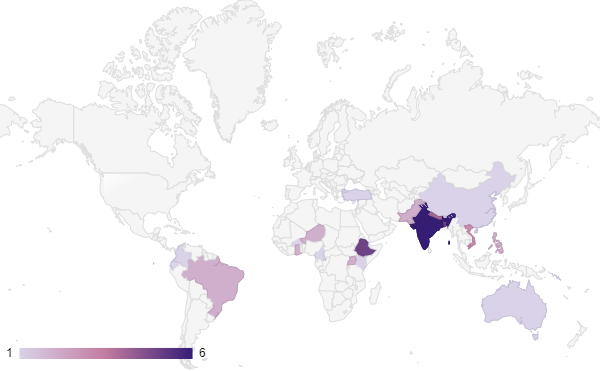  (j) Risk perception |
| 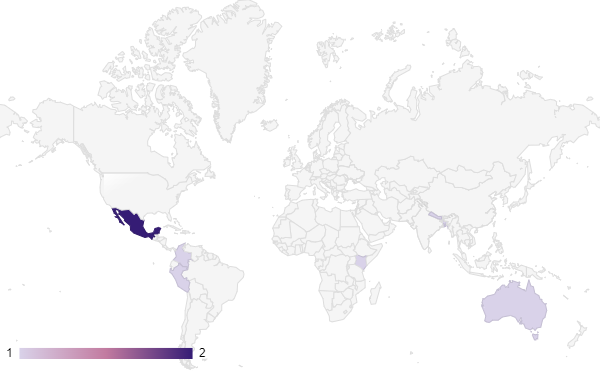  (k) Worldview |  |
